# Integrating bulk, single-cell, and spatial transcriptomics to identify a novel pyroptosis-related gene signature for predicting prognosis and tumor immune landscape in triple-negative breast cancer

**DOI:** 10.3389/fimmu.2026.1743222

**Published:** 2026-04-07

**Authors:** Lingyan Xiang, Jie Rao, Aolong Ma, Yuqi Zhang, Chunlin Li, Ting Xie, Haochen Xue, Zhengzhuo Chen, Tian Liu, Jingping Yuan, Honglin Yan

**Affiliations:** Department of Pathology, Renmin Hospital of Wuhan University, Wuhan, China

**Keywords:** bulk and single-cell RNA sequencing, pyroptosis, pyroptosis-related genes, spatial transcriptomics, triple-negative breast cancer, tumor immune microenvironment

## Abstract

**Introduction:**

Triple-negative breast cancer (TNBC) features significant heterogeneity and a complex tumor immune microenvironment (TIME). Pyroptosis strongly influences this environment, yet the roles of pyroptosis-related genes (PRGs) remain unclear. Since single transcriptomic methods obscure the full clinical value of PRGs, multi-omics identification of PRGs in TNBC was used to predict the prognosis and immune landscape of TNBC.

**Methods:**

We integrated TNBC transcriptomic data from the TCGA and GEO databases. We constructed a PRG prognostic signature using the LASSO algorithm. This signature was validated in an independent GEO cohort. We used single-cell RNA sequencing (scRNA-seq) to analyze the expression heterogeneity of signature genes across different cell subpopulations. We also evaluated their association with the TIME. Spatial transcriptomics (ST) was used to map the spatial distribution of these genes. Finally, we performed immunohistochemistry (IHC) on 48 clinical TNBC samples. This step validated the protein expression of six core genes (PINK1, GZMB, PFKFB3, RSPO3, TREM1, and VEGFA) .

**Results:**

The PRG signature demonstrated robust prognostic predictive performance. It effectively distinguished TNBC patients with different prognoses and immune phenotypes. ScRNA-seq analysis revealed a predominant enrichment of signature genes in T cells. Pseudotime trajectory analysis delineated a continuous T-cell state transition landscape characterized by progressive GZMB upregulation. Cell communication analysis indicated extensive interactions between T cells and macrophages. This interaction occurred via the MIF-CD74-CXCR4 axis. ST confirmed significant expression of signature genes in immune cell enriched regions. IHC results showed that high GZMB and RSPO3 expressions correlated with lower recurrence risk and favorable survival outcomes. Conversely, elevated PINK1, PFKFB3, TREM1, and VEGFA predicted higher recurrence and poorer survival.

**Conclusion:**

We developed a reliable PRG prognostic signature for TNBC. The signature genes demonstrate significant cellular and spatial heterogeneity within the TIME. They drive interactions between T cells and macrophages through the MIF pathway to remodel the TIME. This signature robustly predicts clinical outcomes for patients. It also offers tremendous translational value by providing promising targets for personalized treatment.

## 1 Introduction

Breast cancer (BC) has emerged as the leading cancer by global incidence, threatening the health of women globally (1).Compared to other molecular classifications of BC, triple-negative breast cancer (TNBC) has strong invasiveness, high mortality rate, susceptibility to recurrence and metastasis, and poor prognosis, and has always been a challenging issue in the domain of BC diagnosis and treatment (2). Despite the advancements achieved in TNBC treatment in recent years, a substantial proportion of patients remains fail to reap benefits from neoadjuvant chemotherapy (NAC) or immune checkpoint inhibitors (ICIs) therapy (3, 4). Hence, it is imperative to identify novel and suitable biomarkers to construct pyroptosis-related gene signature (PRG signature) to forecast clinical outcomes and treatment efficacy in TNBC, so as to select the optimal treatment method to improve patient prognosis.

Tumor immune microenvironment (TIME) is a critical regulator of oncogenesis, fundamentally governing malignant processes including tumor cell proliferation, local invasion, and metastatic dissemination. Moreover, it serves as a key determinant of therapeutic efficacy and clinical outcomes in cancer patients (5–7). Recently, pyroptosis has garnered significant attention as it is mechanistically linked to TIME. Pyroptosis is a programmed cell death mechanistically characterized by the activation of inflammatory cytokines (8). This process significantly influences the immune response (8). Despite its confinement to a minor fraction of cells, it is capable of triggering an inflammatory response, regulate TIME, and activate a potent the anti-tumor activity of T cells, synergizing with ICIs to increase the anti-tumor effect (8, 9). However, taking its inflammatory nature into account, abnormal pyroptosis might also be associated with the formation of a tumor-supportive microenvironment (10). Tumor cells may utilize the mechanism of pyroptosis to evade the attack of immune system, suppress immune response, and thereby promote their own survival and proliferation (10). Therefore, the heterogeneity of TIME caused by pyroptosis poses a huge challenge for tumor treatment and prognosis. Nevertheless, the intricate roles and predictive value of pyroptosis-related genes (PRGs) in TNBC are still not very clear, and the capacity of pyroptosis to potentially modulate antitumor immune responses of TNBC remains to be further explored. At present, an increasing number of researchers are investigating the interplay between pyroptosis and oncogenesis and therapeutic approaches, such as lung carcinoma (11), liver carcinoma (12), ovarian carcinoma (13), gastric carcinoma (14). Currently, some scholars have begun to explore the impact of pyroptosis on TNBC (15). The function of pyroptosis in solid tumors has attracted growing attention from researchers.

Motivated by growing biological questions and technical innovation, transcriptome analysis has advanced from bulk RNA-sequencing to single-cell RNA sequencing (scRNA-seq) and spatially resolved approaches, encompassing digital spatial profiling and *in situ* sequencing (16). Bulk RNA-seq has been widely used to construct prognostic signatures across cancer types, yet its tissue-level averaging can mask intratumoral heterogeneity and the contributions of specific immune cell population. Recently, the swift advancement of scRNA-seq technology has facilitated high-throughput and high-resolution analysis of transcriptomes at single cell level, offering novel technical support for analyzing heterogeneity within TIME (17, 18). This powerful tool helps to detect rare cell subtypes, understand tumor evolution, and discover new therapeutic targets, thereby opening up new paths for the clinical management of diverse pathologies (19–22). Despite its high resolution in characterizing cellular heterogeneity, scRNA-seq lacks inherent spatial context, thereby failing to capture the architectural organization of cells within tissue and their location-specific functional states. This limitation has motivated the rapid development of spatially resolved transcriptomic techniques. These techniques enable transcriptome-wide profiling while preserving spatial information (23). These approaches not only map gene expression directly in tissue sections but also provide critical insights into tissue microstructure and regional cell-cell communication. Given the aforementioned advantages, a growing number of studies are integrating two or more transcriptomic technologies to investigate the heterogeneity of TIME and intercellular communication more accurately and comprehensively across multiple dimensions (24–27).

In this study, we established a novel PRG signature for TNBC, which demonstrated robust prognostic accuracy and effectively stratified patients into distinct risk categories with divergent survival outcomes and immune phenotypes. Moving beyond prediction, our multi-omics approach uncovered the functional underpinnings of this signature. Single-cell transcriptomic analysis revealed that signature genes were predominantly active within T cell subsets, linking pyroptosis to T cell functional state transitions and cytotoxic function. Spatial transcriptomics (ST) further illustrated the spatial heterogeneity of these genes, particularly in immune-enriched regions, highlighting their role in shaping TIME. Importantly, these findings were translated into clinical relevance through IHC validation in TNBC samples, demonstrating translational promise in prognostic assessment and as targets for guided treatment strategies. By integrating bulk-seq, scRNA-seq, and ST with clinical validation, this study establishes a robust prognostic framework while deciphering the mechanistic basis of PRGs in shaping the immune landscape and spatial architecture of TNBC, thereby advancing the prospects for personalized therapeutic interventions.

## 2 Material and methods

### 2.1 Data collection and processing

The bulk RNA-seq data and corresponding clinical profiles of TNBC were obtained from the Cancer Genome Atlas (TCGA) database using UCSC Xena (https://xenabrowser.net/datapages/). There were 88 TNBC samples and 9 para-cancerous samples. The data for 135 samples of the GSE19615 (n=28) and GSE58812 (n=107) projects were retrieved from Gene Expression Omnibus (GEO) database (https://www.ncbi.nlm.nih.gov/geo/, accessed on 1 February 2025). Both TCGA project and GEO projects datasets were combined to construct signature’s training cohort. To mitigate potential technical biases, batch effects were corrected using the remove BatchEffect function from the “limma” package (version, 3.58.1) prior to differential expression analysis. The independently recruited GEO cohort consisted of samples obtained from the GSE21653 (n=90) and GSE25065 (n=64) projects within the GEO database (**Supplementary Table S1**). The spatial transcriptomic data GSM6433611 was obtained from the GEO database (https://www.ncbi.nlm.nih.gov/geo/, accessed on 1 February 2025). To ensure robust cross-platform integration, the expression units, transformations, and preprocessing pipelines were strictly defined for each independent cohort (**Supplementary Table S1**). For the TCGA-TNBC training cohort, RNA-sequencing (RNA-seq) data generated via the Illumina HiSeq platform were retrieved. Gene expression levels were quantified as Transcripts Per Million (TPM) and subsequently subjected to a logarithmic transformation, expressed as log2(TPM + 1). For the GEO cohorts, microarray datasets (GSE58812, GSE19615, GSE21653, and GSE25065) generated via Affymetrix Human Genome arrays were downloaded. The pre-processed expression matrices were directly obtained from the GEO database. As reported by the original studies, these datasets had been normalized utilizing platform-specific algorithms: GSE58812 and GSE25065 via MAS 5.0 normalization; GSE21653 via the Robust Multichip Average (RMA) algorithm with quantile normalization; and GSE19615 via dChip invariant set normalization. We confirmed that all microarray expression matrices were appropriately log2-transformed prior to downstream analyses. Regarding microarray probe-to-gene mapping, platform-specific annotation files were employed to convert probe IDs into official HGNC gene symbols. In instances where multiple probes mapped to a single gene locus, the probe exhibiting the maximum expression value across samples was retained to represent the transcript level of that gene. Finally, patients lacking corresponding survival data or clinical follow-up were excluded from the respective prognostic evaluations.

The crucial genes potentially participating in pyroptosis were retrieved from the Molecular Signatures Database (MSigDB), GeneCards database (HALLMARK, C5: GO) (https://www.genecards.org/), and the prior studies on pyroptosis (28)(**Supplementary Table S2**). The initial retrieval yielded 37, 874, and 43 candidate genes from MSigDB, GeneCards, and the literature, respectively. Following the exclusion of shared genes, 885 pyroptosis genes were included.

Transcriptome data were processed with “limma” package, and differentially expressed genes (DEGs) were categorized according to false discovery rate (FDR)< 0.05 and |log2 FC| > 1. A Venn diagram was created with the help of the “VennDiagram” package (version, 1.7.3). DEGs’expression was depicted using volcano plots, with the “ggplot2” package (version, 4.0.1). DEGs’expression heatmaps were produced using “pheatmap” package (version, 1.0.12). To evaluate the impact of the inherent sample size imbalance between tumor (n=88) and normal (n=9) tissues in the TCGA-TNBC cohort, a sensitivity analysis was performed. We compared the DEGs identified from the integrated dataset with those derived solely from the TCGA-TNBC cohort to ensure that the gene discovery process was robust and not driven by the limited number of normal samples.

IHC analysis of 48 TNBC TMAs from Renmin Hospital of Wuhan University was used to further validate the gene expression. All participants provided written informed consent, and the study received approval from the Ethics Committee for Scientific Research at Renmin Hospital of Wuhan University.

### 2.2 Construction of pyroptosis-related signature

We removed patients without survival information from the cohort used for signature construction, leaving 193 patients for subsequent survival analysis. For the training cohort, the primary endpoint was OS. For the GEO cohort, due to the immaturity of overall survival (OS) data in the specific datasets used, disease-free survival (DFS) was adopted as the endpoint. A prognostic gene signature was developed using Lasso regression implemented through the “glmnet” (version, 4.1-8) and “survival” (version, 3.8-3) packages in R. This machine learning approach was specifically selected due to its dual capability of feature selection and regularization, which is particularly advantageous for analyzing high-dimensional genomic data. During the PRG signature development, we systematically determined optimal penalty parameter (λ) through cross-validation to establish gene-specific coefficients for the risk score calculation. The final risk prediction signature was derived from the training cohort using the following formulation:


$$Riskscore=\sum_{i=1}^{N}\left(exp_{i}\times coef_{i}\right)$$

The prognostic risk score was determined using the following formula: for each patient, the total of each value multiplied by gene expression values (exp) and their corresponding regression coefficients (coef) across N selected genes was computed. Based on this risk score, patients in training cohort were stratified into high- and low-risk categories. Differences in survival between these groups were evaluated through Kaplan-Meier estimation, which effectively displays temporal survival patterns and facilitates interpretation of prognostic outcomes. To quantify the ability to predict outcomes of our signature, time-dependent receiver operating characteristic (ROC) analysis was conducted with the “timeROC” package (version, 0.4), which measures the trade-off between sensitivity and specificity at various time points.

### 2.3 Consensus clustering analysis of the signature genes

Using “ConsensusClusterPlus” package (version, 1.66.0), TNBC patients (n=223) in TCGA cohort were stratified into two distinct molecular subgroups based on signature genes expression patterns. The optimal number of clusters (k=2) was confirmed through cumulative distribution function (CDF) analysis.

### 2.4 Functional enrichment analysis

To elucidate the biological significance and signaling pathways associated with the risk stratification, we performed enrichment analyses based on the DEGs identified between the high- and low-risk groups. The ‘clusterProfiler’ package (version 4.10.1) in R was utilized to conduct Gene Ontology (GO) annotation and Kyoto Encyclopedia of Genes and Genomes (KEGG) pathway enrichment analyses.

### 2.5 TIME analysis

We performed ssGSEA to evaluate immune cell infiltration and functional activity in TIME using “limma”, “GSVA” (version, 1.50.5), and “GSEABase” (version, 1.64.0) packages. Data processing and visualization were conducted with “reshape” and “ggpubr”. Using “pheatmap”, we generated heatmaps to display associations between signature genes, risk scores, and immune features. TIME composition was analyzed with the “estimate” package (version, 1.0.13), including immune score comparisons between clusters. This methodology enabled the quantification of both immune pathway activation and the extent of immune cell infiltration. Moreover, we applied “CIBERSORT” (version, 0.1.0) to assess the proportions of 22 distinct tumor-infiltrating immune cell populations in high- versus low-risk groups. To ensure a comprehensive and unbiased representation of the immune landscape, all samples were included in the downstream analysis without p-value-based filtering. We further investigated the correlations between signature genes and specific immune cell subtypes, as well as associations with key immune checkpoint molecules.

### 2.6 Single-cell analysis

ScRNA-seq data were processed using Seurat pipeline, including quality control, normalization, and unsupervised clustering. Low-quality cells were excluded based on quality control criteria: cells with nFeature_RNA< 500 or > 6000, nCount_RNA< 1000 or > 50000, or mitochondrial gene content (percent.mt)< 1% or > 20% were removed. Subsequently, potential doublets were identified and removed using the “scDblFinder” package (version, 1.16.0). Clustering was conducted at a resolution of 0.5 using the top 20 dimensions. Dimensionality reduction was carried out using uniform manifold approximation and projection (UMAP), followed by celltype annotation using marker genes identified through the FindAllMarkers function (min.pct = 0.25, logFC threshold = 0.25, p< 0.05). T cell subsets (CD8^+^, CD4^+^, and Tregs) were classified based on canonical markers curated from literature.

### 2.7 Signature genes activity scoring

Using the 6 identified signature genes, we calculated signature scores for all cells via the AddModuleScore function. Cells with scores exceeding the threshold (score > 0.3) were defined as signature-high. Their spatial distribution across clusters was visualized on UMAP plots, highlighting activated populations.

### 2.8 Pseudotime trajectory analysis

T cell clusters were isolated and subclassified by marker genes. Using Monocle’s DDRTree algorithm, we reconstructed transcriptional trajectories to order cells along a continuum of functional states. This analysis was used to characterize transcriptional state transitions within T-cell subsets rather than inferred lineage differentiation. The dynamics of signature gene activity were then visualized across the pseudotime trajectory.

### 2.9 Cell-cell communication

Cell-cell interactions were inferred using the “CellChat” package (version, 1.6.1) with the CellChatDB.human database. Intercellular communication networks were systematically analyzed using the “CellChat” package to identify and quantify significant ligand-receptor interactions between cell populations.

### 2.10 Spatial transcriptome analysis

ST data processing and visualization were performed using an integrated analytical pipeline incorporating the “Seurat” (version, 5.3.0), “dplyr” (version, 1.1.4), “patchwork” (version, 1.3.2) and “ggplot2” packages. The 10x Visium ST data (GSM6433611) were analyzed using “Seurat” (version, 5.3.0). Quality control was performed to filter out low-quality spots, retaining those with 200< nFeature_Spatial< 6000 and< 10% mitochondrial gene counts. Data were normalized and scaled using the SCTransform workflow. Dimensionality reduction and unsupervised clustering were conducted using the first 15 principal components. For cell-type annotation, we utilized the robust cell type decomposition (RCTD) method from the spacexr package (v2.2.1) to integrate the single-cell reference dataset with the spatial data. A reference object was constructed using raw counts from the annotated scRNA-seq object. The RCTD algorithm was run in “doublet” mode to allow for the identification of up to two cell types per spatial spot. The resulting primary cell type assignments (first type) were projected onto the spatial tissue coordinates for visualization. Spatial gene expression patterns were subsequently visualized using the SpatialFeaturePlot function, enabling topographic mapping of transcriptional activity.

### 2.11 TNBC patient specimens and immunohistochemical evaluation

Tissue microarray (TMA) samples were obtained from 48 TNBC patients at Renmin Hospital of Wuhan University for IHC analysis. Comprehensive clinicopathological and follow-up data were available for all cases, including age, lymph node metastasis status, histological grade, tumor stage, recurrence information, and 5-year disease-free survival (5-DFS) outcomes. These data allowed for the evaluation of the association between the expression levels of signature genes and clinical prognosis. TMA sections were first treated with xylene to remove paraffin, followed by rehydration through a graded series of ethanol. Following standard protocols, endogenous peroxidase activity was quenched by treating the sections with 3% H_2_O_2_ at room temperature for 10 minutes. Subsequent antigen retrieval was conducted using microwave heating in 10 mM citrate buffer (pH 6.0). Finally, the slides were blocked with goat serum diluted 1:10 for 30 minutes at room temperature. Following antigen retrieval, sections were subjected to overnight incubation at 4 °C with primary antibodies targeting PINK1 (rabbit, 1:300, Proteintech (Wuhan, China), 23274-1-AP), PFKFB3 (rabbit, 1:300, Proteintech (Wuhan, China), 13763-1-AP), RSPO3 (rabbit, 1:200, Proteintech (Wuhan, China), 17193-1-AP), TREM-1 (rabbit, 1:200, Proteintech (Wuhan, China), 11791-1-AP), GZMB (rabbit, 1:200, Proteintech (Wuhan, China), 13588-1-AP) and VEGFA (mouse, 1:4000, Proteintech (Wuhan, China), 19003-1-AP). After washing with PBS, the sections were incubated with broad-spectrum HRP-conjugated secondary antibodies for 30 minutes at room temperature. Nuclear staining was visualized with hematoxylin, and whole-slide images were acquired using an Olympus bright-field microscope (Olympus Corporation, Tokyo, Japan).

IHC score was determined by evaluating both staining intensity and proportion of tumor cells exhibiting positive signals, using the formula IHC score = intensity score × percentage score. The scoring standards were defined as follows: percentage of positive cells, 0 (<10%), 1 (10–25%), 2 (26–50%), 3 (51–75%), and 4 (>75%); staining intensity, 0 (no staining), 1 (light brown), 2 (brown), and 3 (dark brown). Based on the total IHC score, TNBC samples were classified into positive and negative expression groups for each gene. Cases with an IHC score greater than 4 were considered positive, while those with a score of 4 or less were regarded as negative for PINK1, PFKFB3, TREM1, GZMB, RSPO3, and VEGFA.

### 2.12 Statistical analysis

Data were processed and analyzed using R version 4.3.2 (2023-10-31), SPSS 27.0, along with Zstats 1.0 (www.zstats.net). For continuous variables, values are reported as mean ± standard error and examined with either Student’s t-test or the Wilcoxon rank-sum test. Survival curves were estimated using Kaplan–Meier method, and differences between groups were assessed with log-rank test. We used chi-square test to analyze categorical data. Chi-square analysis was conducted to assess the association between tumor recurrence and clinicopathological parameters. Spearman’s correlation was applied to investigate the association between tumor recurrence and clinicopathological characteristics. Furthermore, logistic regression was utilized to determine factors associated with tumor recurrence. Statistical significance was defined as a p-value less than 0.05. To rigorously evaluate the independent prognostic value of the risk signature beyond conventional immune metrics, a comprehensive multivariable Cox proportional hazards regression model was constructed utilizing the well-annotated TCGA-TNBC cohort. This model incorporated the signature risk score alongside age, clinical stage, CD8A expression (as a surrogate for CD8^+^ T cell infiltration), and the Cytolytic Activity (CYT) score. The CYT score was computed as the arithmetic mean of the log2-transformed expression values of GZMA and PRF1. Patients lacking complete clinical parameters were excluded from this specific multivariable analysis. Due to the relatively small number of clinical events in the TCGA-TNBC cohort, the multivariable Cox analysis was interpreted with caution regarding potential over-fitting and wide confidence intervals.

## 3 Results

### 3.1 Identification of prognostic PRGs in TNBC

**Figure 1:**
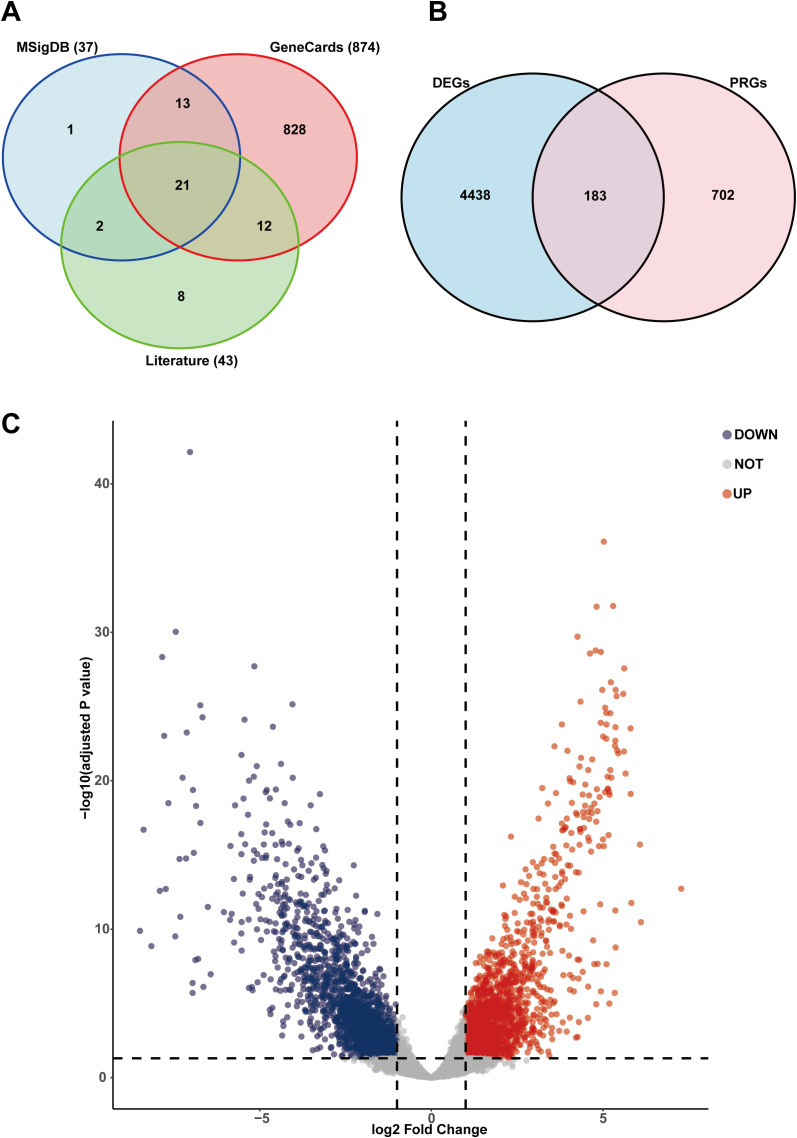
Identification of PRGs in TNBC based on transcriptomics. **(A)** Venndiagram indicates 885 PRGs from MSigDB, GeneCards, and literature. **(B)** Venndiagram indicates 183 DE-PRGs between PRGs and the DEGs of bulk transcriptome. **(C)** Volcanic map for all differentially expressed genes identified by transcriptome sequencing in normal versus tumor groups. Blue and red dots denote genes downregulated and upregulated in tumors, respectively.

Based on transcriptomic expression profiles from TNBC and normal breast tissues, expression data of PRGs were selectively extracted for further investigation. Differential expression analysis was performed on 223 TNBC samples and 9 adjacent normal tissue samples. Analysis of differential expression revealed a total of 4510 DEGs between TNBC and normal samples. The initial compilation from MSigDB (37 genes), GeneCards (874 genes), and literature (43 genes) yielded 885 unique PRGs after deduplication (**Figure 1A**). By intersecting these DEGs with a set of 885 known PRGs, 183 differentially expressed pyroptosis-related genes (DE-PRGs) were obtained (**Figure 1B**). We used volcano plots to visualize all differentially expressed genes. (**Figure 1C**). Furthermore, to address the potential bias introduced by the inherent sample size imbalance in the TCGA cohort (88 tumor vs. 9 normal samples), we performed a dedicated sensitivity analysis restricted to the TCGA-TNBC dataset. The results demonstrated an exceptionally high concordance, with 166 out of the 183 (90.71%) primary DE-PRGs remaining statistically significant with identical expression directions. This high level of stability confirms that the biological signals captured by the identified DE-PRGs are intrinsic to TNBC pathology rather than an artifact resulting from normal-sample sourcing imbalance.

### 3.2 Development and verification of a PRG signature

**Figure 2:**
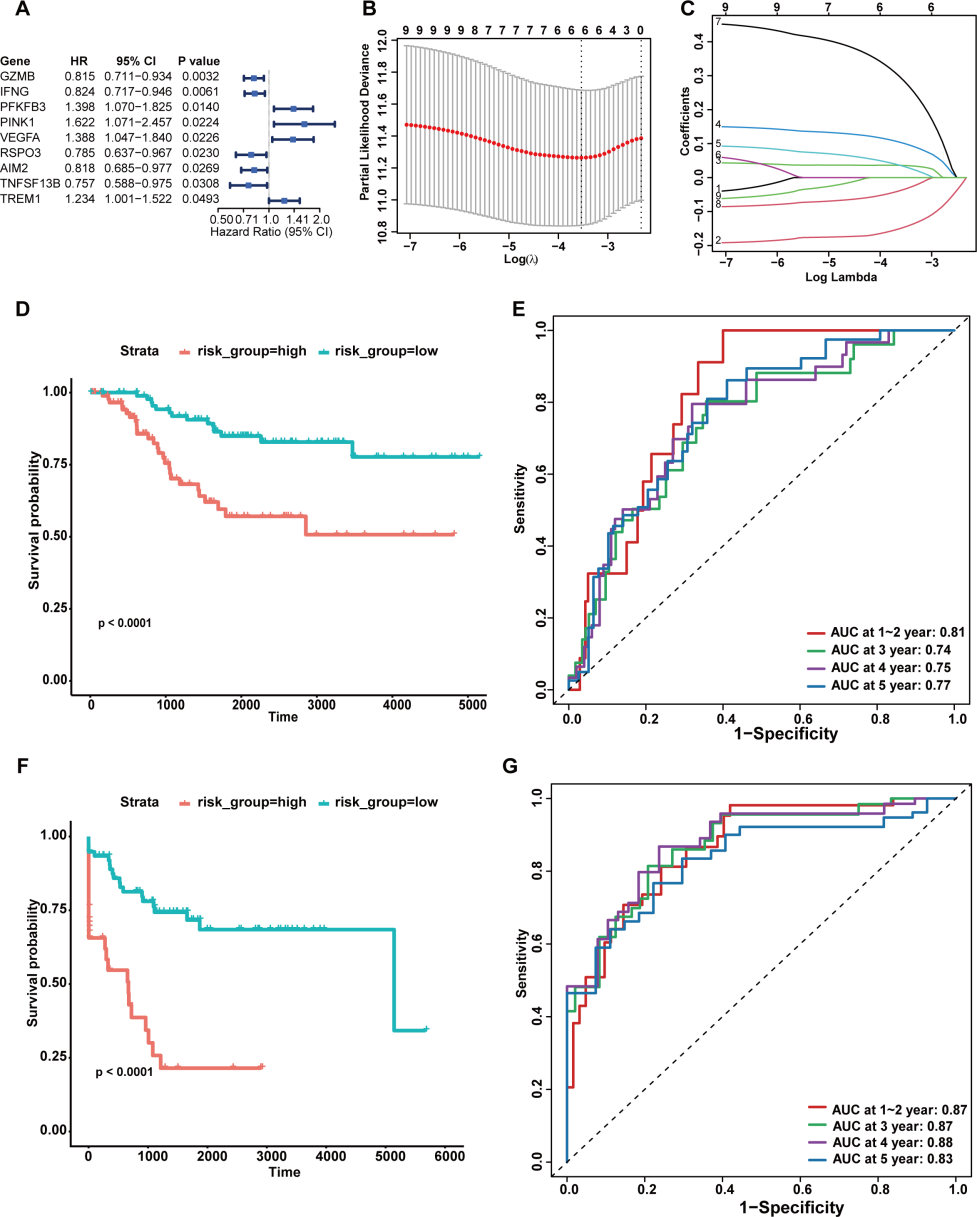
Construction and performance of the PRG signature in distinct cohorts. **(A)** Forest plot of univariate cox regression analysis of DE-PRGs. **(B, C)** The selection of signature genes based on the optimal parameter λ that was obtained in the LASSO regression analysis. **(D)** K-M curve displayed survival outcomes of patients in two risk groups from signature training cohort. **(E)** A time-dependent ROC curve was drawn to assess survival rate at 1–2 year, 3 year, 4 year, and 5-year in signature training cohort. **(F)** K-M curve illustrate the survival outcomes of the two risk groups within the GEO cohort. **(G)** A time-dependent ROC curve was drawn toassess survival rate at 1–2 year, 3 year, 4 year, and 5-year in GEO cohort. Statistical significance for K-M curves was determined using the log-rank test. P-values in ROC analyses represent the AUC significance.

We extracted the clinical characteristics of 193 TNBC patients with complete survival information from the TCGA-TNBC and GSE58812 datasets. We assessed batch effects using PCA analyses before and after batch correction. As shown in **Supplementary Figure S1**, samples were primarily separated by data source before correction, whereas samples from different datasets were well mixed after correction while preserving tumor-normal differences. These analyses confirm that batch effects were adequately mitigated. Univariate Cox regression analysis was used to screen DE-PRGs and 9 genes (GZMB, IFNG, PFKFB3, PINK1, VEGFA, RSPO3, AIM2, TNFSF13B and TREM1) were significantly correlated with the prognosis of TNBC (**Figure 2A**). We performed LASSO regression to narrow down the candidate genes. Ultimately, 6 signature genes—PINK1, GZMB, PFKFB3, RSPO3, TREM1, and VEGFA—were retained to construct a PRG signature based on the optimal lambda value (**Figures 2B, C**). The risk score for each patient was calculated using the following formula, where each gene symbol represents its mRNA expression level: Risk score = (-0.158360246047182) × GZMB + 0.32235503219597 × PINK1 + 0.111243442274487 × PFKFB3 + (-0.0464626296260152) × RSPO3 + 0.0437384204335007 × TREM1 + 0.0364743584502037 × VEGFA. Stratification of the training cohort patients into high- and low-risk subgroups was accomplished based on the median risk score. Kaplan-Meier curve (K-M curve) (**Figure 2D**) showed that patients in high-risk group exhibited markedly worse OS than those in low-risk group (p< 0.0001). we performed ROC curve analysis to evaluate the predictive performance of the signature (**Figure 2E**). The area under the curve (AUC) values were 0.81 at 1–2 years, 0.74 at 3 years, 0.75 at 4 years, and 0.77 at 5 years, indicating the excellent prognostic accuracy of the signature in predicting OS. To assess the generalizability of the signature, an independent GEO cohort consisting of the GSE21653 and GSE25065 datasets was analyzed. As expected, the signature exhibited robust predictive power in GEO cohort, with high-risk group showing significantly reduced DFS compared to low-risk group (p< 0.0001, **Figure 2F**). The AUC values for 1–2, 3, 4, and 5 years were 0.87, 0.87, 0.88, and 0.83, respectively (**Figure 2G**). In conclusion, the risk scoring signature derived from Lasso–Cox regression exhibited robust prognostic performance across distinct clinical endpoints, effectively predicting OS in the training cohort and successfully extending its relevance to predict DFS in the independent GEO cohort, providing a potential tool for forecasting patient prognosis in TNBC. Additionally, we performed a sensitivity analysis using a curated pyroptosis-related gene set. A significant negative correlation was observed between the risk score and the activity of the canonical pyroptosis pathway assessed by ssGSEA (Spearman’s ρ = –0.415, p = 2.76 × 10^-9^) (**Supplementary Figure S2**). This indicates that patients with higher activation of the pyroptosis-related pathway tend to have lower risk scores. To further rigorously validate the superiority of our gene selection strategy, we performed a sensitivity analysis using 27 canonical core pyroptosis genes in the training cohort (**Supplementary Figure S3**). The prognostic model derived strictly from these core genes exhibited limited predictive value (C-index = 0.500), whereas our proposed broad-spectrum PRG signature achieved a significantly higher C-index of 0.722. This distinct advantage suggests that while core genes are involved in the execution machinery of cell death, the broader regulatory network captured by our signature offers superior prognostic stratification in TNBC. Furthermore, the functional association of these six signature genes with pyroptosis has been well-documented in previous studies (29–34), reinforcing the biological plausibility of our prognostic model. Recognizing the inherent challenges of cross-platform data integration and the potential for platform-specific noise, we conducted a rigorous sensitivity analysis to validate the robustness of our signature. We stratified the combined training cohort back into its constituent single-platform datasets: the TCGA RNA-seq cohort and the GSE58812 microarray cohort. Importantly, the established prognostic signature was evaluated independently within each platform without any further modification. As illustrated in **Supplementary Figure S4**, the signature demonstrated consistent and robust predictive performance across both technologies. Specifically, it achieved an AUC of 0.76 in the TCGA RNA-seq cohort (**Supplementary Figures S4A, C**) and, notably, an even superior AUC of 0.85 (Log-rank P = 0.00028) in the GSE58812 cohort (**Supplementary Figures S4B, D**). This excellent reproducibility confirms that our batch correction strategy effectively mitigated technical artifacts and that the derived signature captures intrinsic biological signals relevant to TNBC prognosis.To further evaluate the robustness of our signature and eliminate potential bias introduced by cohort-specific risk stratification, we assessed the prognostic performance of the risk score as a continuous variable (standardized per 1-SD increase). Univariate Cox regression analysis demonstrated that the continuous risk score remained a powerful predictor in both cohorts. Specifically, in the training cohort (outcome: OS), the Hazard Ratio (HR) was 1.926 per 1-SD increase (95% CI: 1.445–2.567, P< 0.0001). Consistent results were observed in the GEO cohort (outcome: DFS), with an HR of 2.780 per 1-SD increase (95% CI: 1.993–3.880, P< 0.0001). These findings, now presented in **Supplementary Figure S5**, confirm that the risk score is linearly and significantly associated with adverse patient outcomes, validating its intrinsic predictive ability independent of dichotomous cutoffs. Finally, to address the inherent platform-scale differences between the RNA-seq (TCGA) and microarray (GEO) datasets and eliminate the possibility of platform-induced artifacts, we performed an additional comprehensive sensitivity analysis. Alternative data harmonization strategies were employed, including strict within-dataset Z-score normalization prior to dataset merging, as well as an empirical Bayes framework (ComBat). Importantly, the coefficient directions and statistical significance (P< 0.05) of all six signature genes (GZMB, TREM1, RSPO3, VEGFA, PFKFB3, PINK1) remained completely consistent across these distinct preprocessing methods (**Supplementary Figure S6**). These results strongly confirm that our gene selection is highly stable and driven by true biological differences, rather than being influenced by technical batch effects.

### 3.3 Consensus clustering analysis

**Figure 3:**
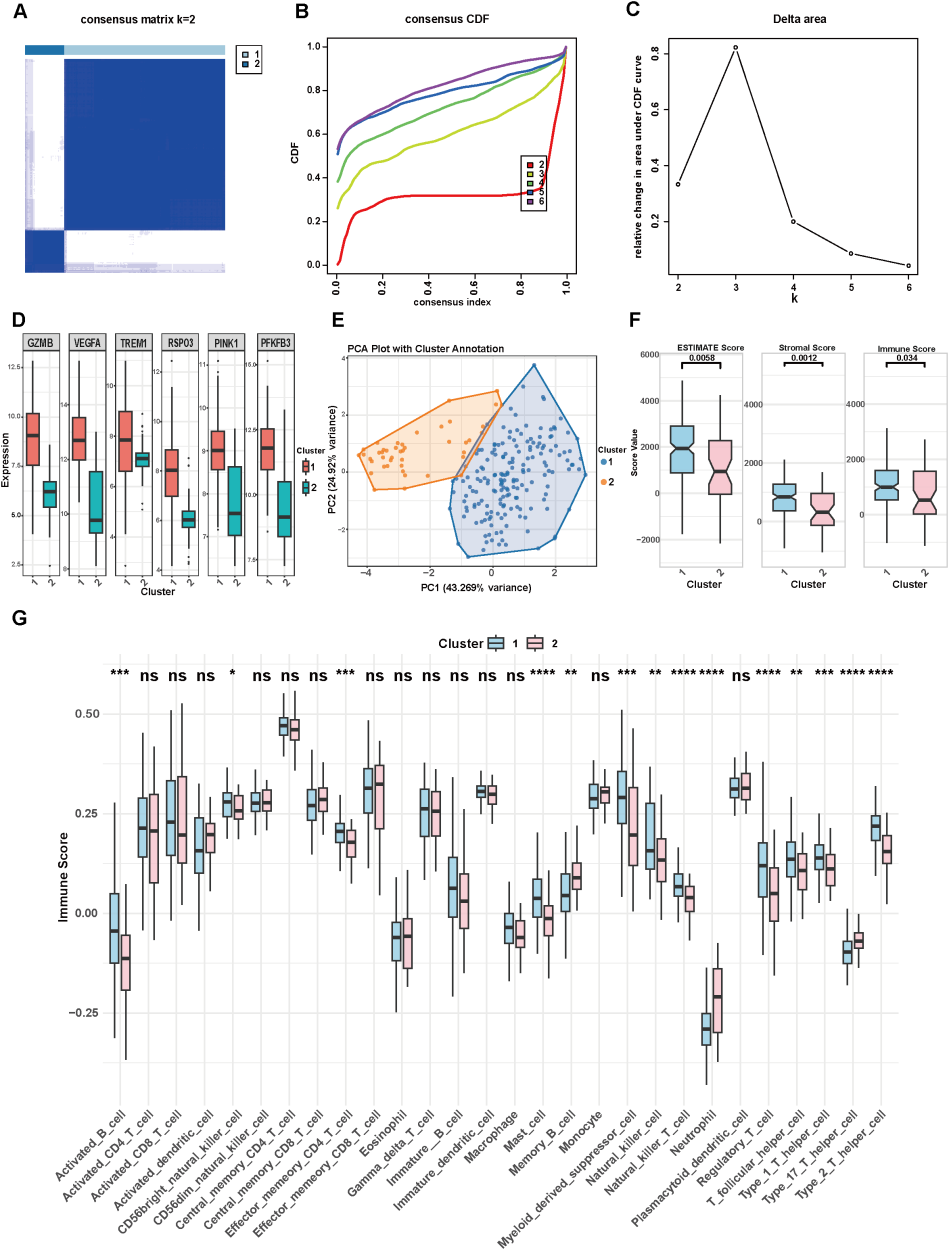
Consensus clustering analysis. **(A)** Heatmap of clustering at consensus k = 2. **(B)** CDF curves of different consensus k-values. **(C)** Delta area under the CDF curve, represents the relative change in area under the CDF curve for each clustering number (k). **(D)** Difference of 6 signature genes expression levels between 2 clusters. **(E)** Principal component analysis of 2 clusters. **(F)** Comparison of immune score between 2 clusters. **(G)** Difference of immune infiltration score between 2 clusters calculated by ssGSEA.*P< 0.05, **P< 0.01, ***P< 0.001, ****P< 0.0001.

To investigate the relationship between 6 signature genes and TNBC subtypes, consensus clustering analysis was performed on all 223 TNBC patients from signature training cohort. The CDF curve, representing the proportion of inter-sample similarity scores at or below a given threshold, was employed to select the optimal cluster number. The best division was achieved when k = 2, as indicated by the CDF curve (**Figures 3A–C**). Accordingly, the 223 TNBC patients were classified into two distinct clusters: 179 samples in Cluster 1 and 44 samples in Cluster 2. We then compared the expression levels of the 6 signature genes between the two clusters (**Figure 3D**). All 6 genes showed significantly higher expression in Cluster 1. Principal component analysis (PCA) additionally validated the clear separation between the two clusters (**Figure 3E**). To explore immune characteristics, ESTIMATE algorithm was implemented to calculate immune, stromal, and ESTIMATE scores for all TNBC samples. The results revealed statistically significant differences in immune scores between the two clusters (**Figure 3F**). Specifically, Cluster 1 exhibited higher ESTIMATE, stromal, and immune scores than Cluster 2. In addition, immune infiltration levels were assessed with the ssGSEA algorithm. Cluster 1 showed markedly elevated infiltration levels of numerous immune cell subsets, such as activated B cells, CD56bright natural killer (NK) cells, effector memory CD4^+^ T cells, myeloid-derived suppressor cells, mast cells, NK cells, NKT cells, Tregs, Th1 cells, T follicular helper cells (Tfh), and Th2 cells (**Figure 3G**). Conversely, Cluster 2 exhibited higher infiltration of memory B cells, neutrophils, and Th17 cells compared to Cluster 1. To further rule out the potential confounding effect of immune infiltration abundance on our prognostic model, we performed a multivariate Cox regression analysis incorporating both the risk score and the Immune Score (calculated via the ESTIMATE algorithm) as covariates. As shown in **Supplementary Figure S7**, the risk score remained a highly significant and independent prognostic factor (HR = 7.183, 95% CI: 2.852–18.092, P< 0.001) even after adjusting for overall immune infiltration intensity. In contrast, the Immune Score itself did not demonstrate statistical significance in the multivariate model (P = 0.138). These findings indicate that the prognostic predictive power of our signature is not merely a reflection of immune cell abundance but captures intrinsic biological features of the tumor that are closely associated with patient outcomes. However, we acknowledge that the relatively wide confidence intervals in this multivariable model may reflect the limited number of clinical events in the TCGA-TNBC cohort. Therefore, these findings regarding independent prognostic value should be interpreted with caution and require further validation in larger, prospective studies.

### 3.4 Functional enrichment analysis of high-risk group

**Figure 4:**
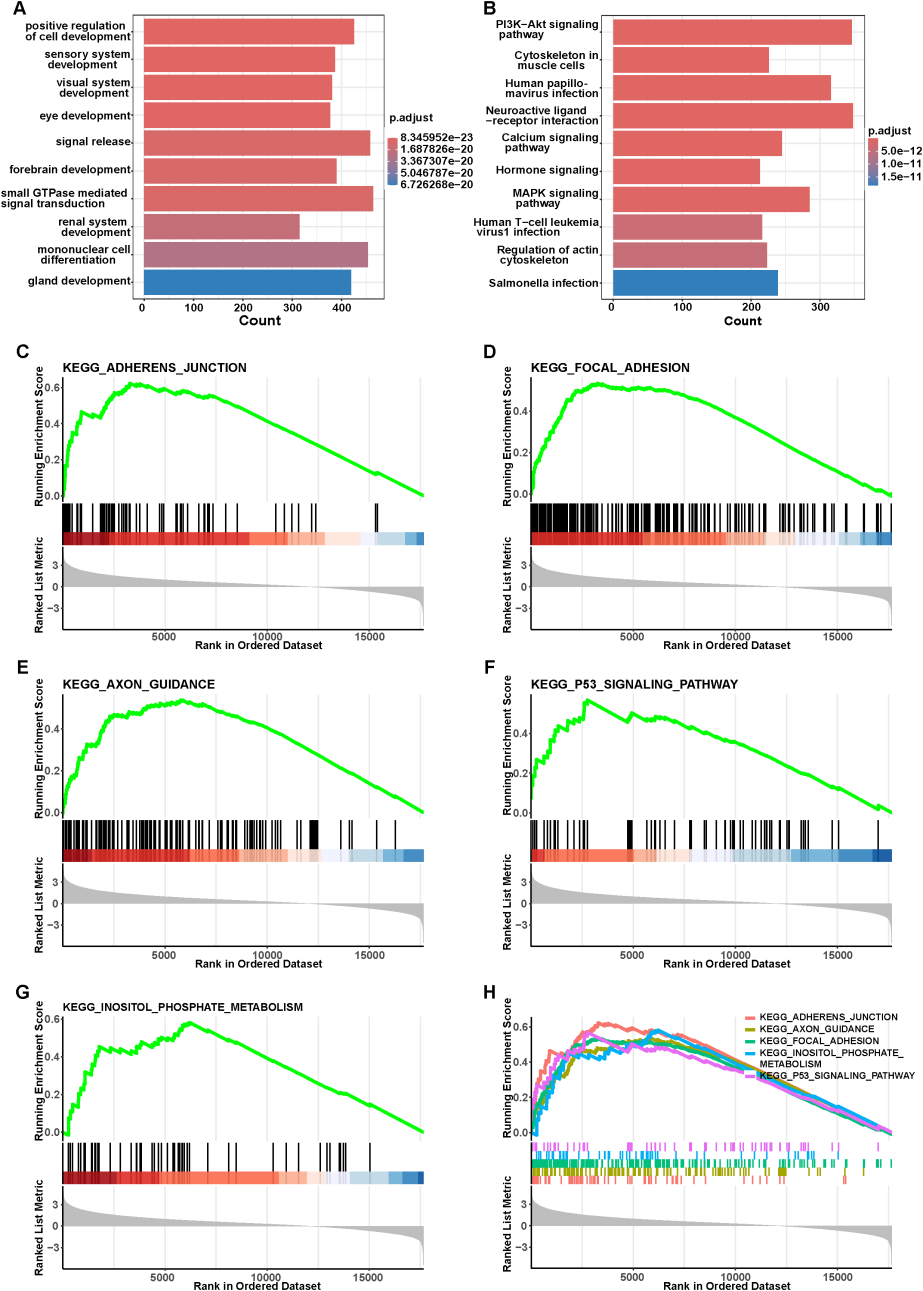
Functional enrichment analysis of the high-risk group. **(A)** GO analysis based on DEGs between high- and low-risk groups. **(B)** KEGG analysis based on DEGs between high- and low-risk groups. **(C–H)** Highly expressed GSEA pathway in high-risk group.

The high- and low-risk groups, stratified based on the PRG signature, underwent functional and pathway enrichment analysis. GO enrichment analysis revealed that high-risk group was predominantly associated with biological processes related to development and cell differentiation (**Figure 4A**). Consistently, KEGG pathway analysis identified that several classical cancer-related and immune-associated pathways were significantly enriched in the high-risk group (**Figure 4B**). These included PI3K-Akt signaling pathway, calcium signaling pathway, and MAPK signaling pathway. To further investigate the molecular mechanisms underlying the prognostic gene signature, GSEA was performed based on the risk score. Among the remarkably enriched pathways, five KEGG pathways were identified in high-risk group, including adherens junction (**Figure 4C**), focal adhesion (**Figure 4D**), axon guidance (**Figure 4E**), the p53 signaling pathway (**Figure 4F**), and inositol phosphate metabolism (**Figure 4G**). These pathways are mainly associated with cell adhesion, migration, intracellular signaling, and tumor suppressor responses, suggesting their potential involvement in the malignant progression observed in high-risk patients.

### 3.5 Immune infiltration analysis

**Figure 5:**
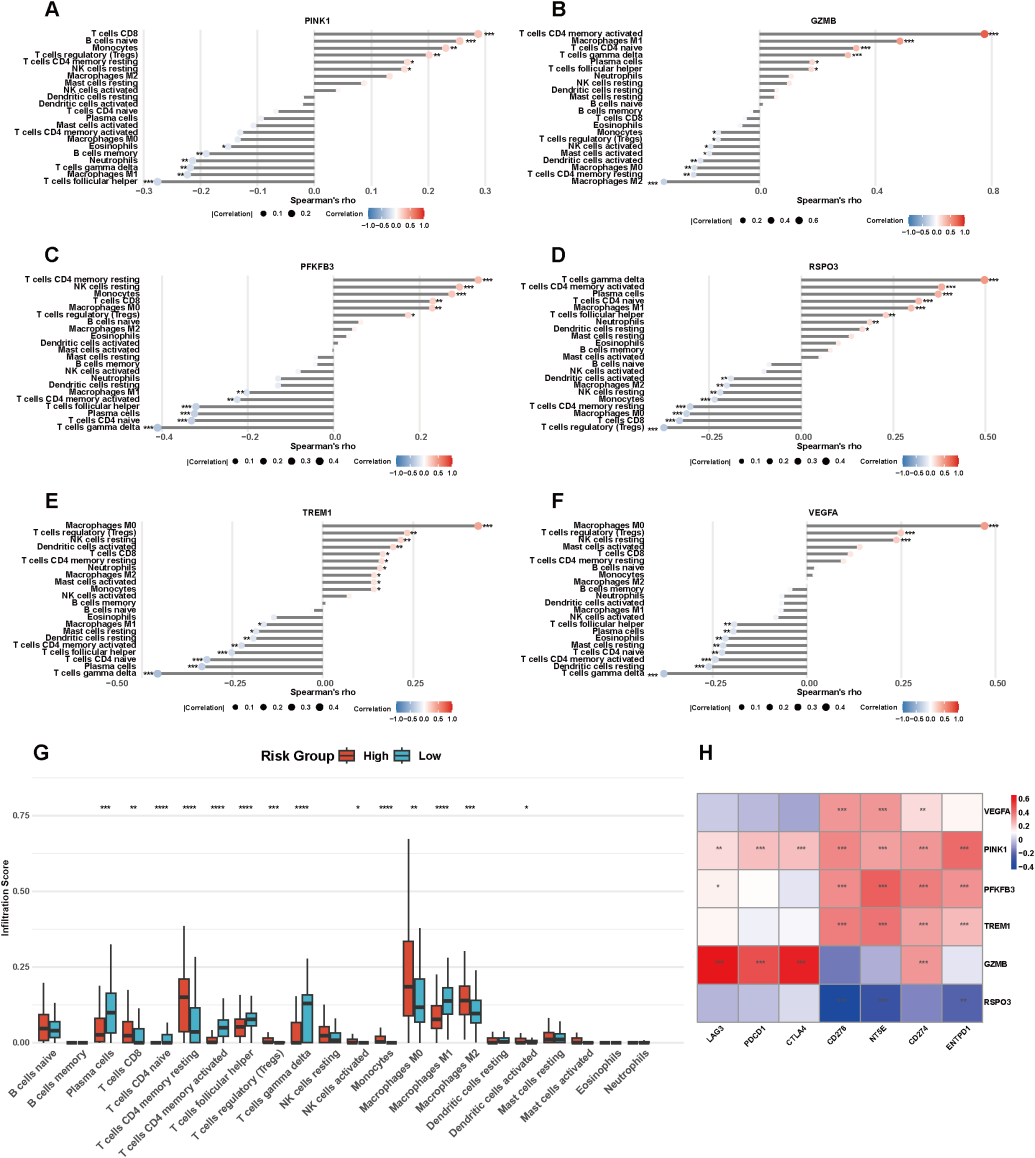
Immune infiltration analysis. **(A)** Correlation analysis between PINK1 and immune cells. **(B)** Correlation analysis between GZMB and immune cells. **(C)** Correlation analysis between PFKFB3 and immune cells. **(D)** Correlation analysis between RSPO3 and immune cells. **(E)** Correlation analysis between TREM1 and immune cells. **(F)** Correlation analysis between VEGFA and immune cells. **(G)** Difference of immune infiltration between high- and low-risk groups. **(H)** Correlation analysis between signature genes (PINK1, GZMB, PFKFB3, RSPO3, TREM1 and VEGFA) and immune checkpoints. *P< 0.05, **P< 0.01, ***P< 0.001, ****P< 0.0001.

To investigate the interrelation between signature genes and TIME, the relative abundances of 22 tumor-infiltrating immune cell populations in high- and low-risk groups were assessed using CIBERSORT. PINK1 expression was positively correlated with naive B cells, CD8^+^ T cells, monocytes, Tregs, resting memory CD4^+^ T cells, and resting NK cells. A significant negative correlation was observed with Tfh, M1 macrophages, γδ T cells, neutrophils, memory B cells, and eosinophils (**Figure 5A**). GZMB showed positive correlations with activated memory CD4^+^ T cells, M1 macrophages, naive CD4^+^ T cells, plasma cells, γδ T cells, and Tfh, while exhibiting negative associations with M2 macrophages, M0 macrophages, resting memory CD4^+^ T cells, activated dendritic cells (DCs), activated mast cells, activated NK cells, Tregs, and monocytes (**Figure 5B**). PFKFB3 showed a positive association with resting NK cells, CD8^+^ T cells, resting memory CD4^+^ T cells, monocytes, M0 macrophages, and Tregs, but negatively correlated with γδ T cells, plasma cells, naive CD4^+^ T cells, Tfh, activated memory CD4^+^ T cells, and M1 macrophages (**Figure 5C**). RSPO3 positively correlated with activated memory CD4^+^ T cells, γδ T cells, plasma cells, naive CD4^+^ T cells, M1 macrophages, neutrophils, Tfh, and resting DCs, while negatively associated with Tregs, M0 macrophages, CD8^+^ T cells, monocytes, resting NK cells, resting memory CD4^+^ T cells, M2 macrophages, and activated DCs (**Figure 5D**). TREM1 demonstrated a positive correlation with M0 macrophages, Tregs, resting NK cells, activated DCs, CD8^+^ T cells, resting memory CD4^+^ T cells, neutrophils, M2 macrophages, activated mast cells, and monocytes, while negatively correlated with γδ T cells, plasma cells, naive CD4^+^ T cells, Tfh, activated memory CD4^+^ T cells, resting DCs, resting mast cells, and M1 macrophages (**Figure 5E**). VEGFA showed positive correlations with M0 macrophages, Tregs, and resting NK cells, but was negatively associated with γδ T cells, resting DCs, activated memory CD4^+^ T cells, naive CD4^+^ T cells, resting mast cells, eosinophils, plasma cells, and Tfh (**Figure 5F**). A comparative analysis of immune cell infiltration was conducted between high- and low-risk groups (**Figure 5G**). The high-risk group exhibited significantly increased infiltration of CD8^+^ T cells, resting memory CD4^+^ T cells, Tregs, activated NK cells, monocytes, M0 macrophages, M2 macrophages, and activated DCs. In contrast, activated memory CD4^+^ T cells, Tfh, plasma cells, γδ T cells, and M1 macrophages exhibited significantly lower abundance in the high-risk cohort. Finally, we assessed the correlations between DE-PRGs and seven widely studied immune checkpoint genes (CD276, CTLA4, ENTPD1, LAG3, NT5E, PDCD1, CD274) (**Figure 5H**). RSPO3 was negatively correlated with CD276, NT5E, and ENTPD1. GZMB showed positive correlations with LAG3, PDCD1, CTLA4, and CD274. TREM1 was positively associated with CD276, NT5E, CD274, and ENTPD1. PFKFB3 exhibited positive correlations with LAG3, CD276, NT5E, CD274, and ENTPD1. PINK1 was positively correlated with with all seven immune checkpoint genes. VEGFA was positively associated with CD276, NT5E, and CD274. Given that signature genes are strongly immune-associated, we sought to ascertain whether the risk score provides prognostic information beyond conventional clinical characteristics and baseline immune cell cytolytic activity. We performed a comprehensive multivariable Cox regression analysis in the well-annotated TCGA-TNBC cohort. The full model incorporated age, clinical stage, CD8A expression, cytolytic activity (CYT) score, and the risk score. Remarkably, even when competing against these established clinical and immune variables, our risk score remained a highly significant and independent risk factor for overall survival (P = 0.019, HR = 13.31; **Supplementary Figure S8**). Interestingly, neither CD8A expression (P = 0.222) nor the CYT score (P = 0.946) retained statistical significance in this comprehensive model. These findings strongly demonstrate that our signature does not merely reflect baseline immune abundance or cytolytic activity, but rather captures unique, independent, and superior prognostic information regarding the intrinsic aggressiveness of TNBC. However, we acknowledge the relatively small number of events in the TCGA-TNBC cohort, which is reflected in the wide confidence intervals. Further validation in larger independent cohorts is warranted to confirm the stability of this multivariable model.

### 3.6 Single-cell atlas of carcinoma and normal tissues in TNBC

**Figure 6:**
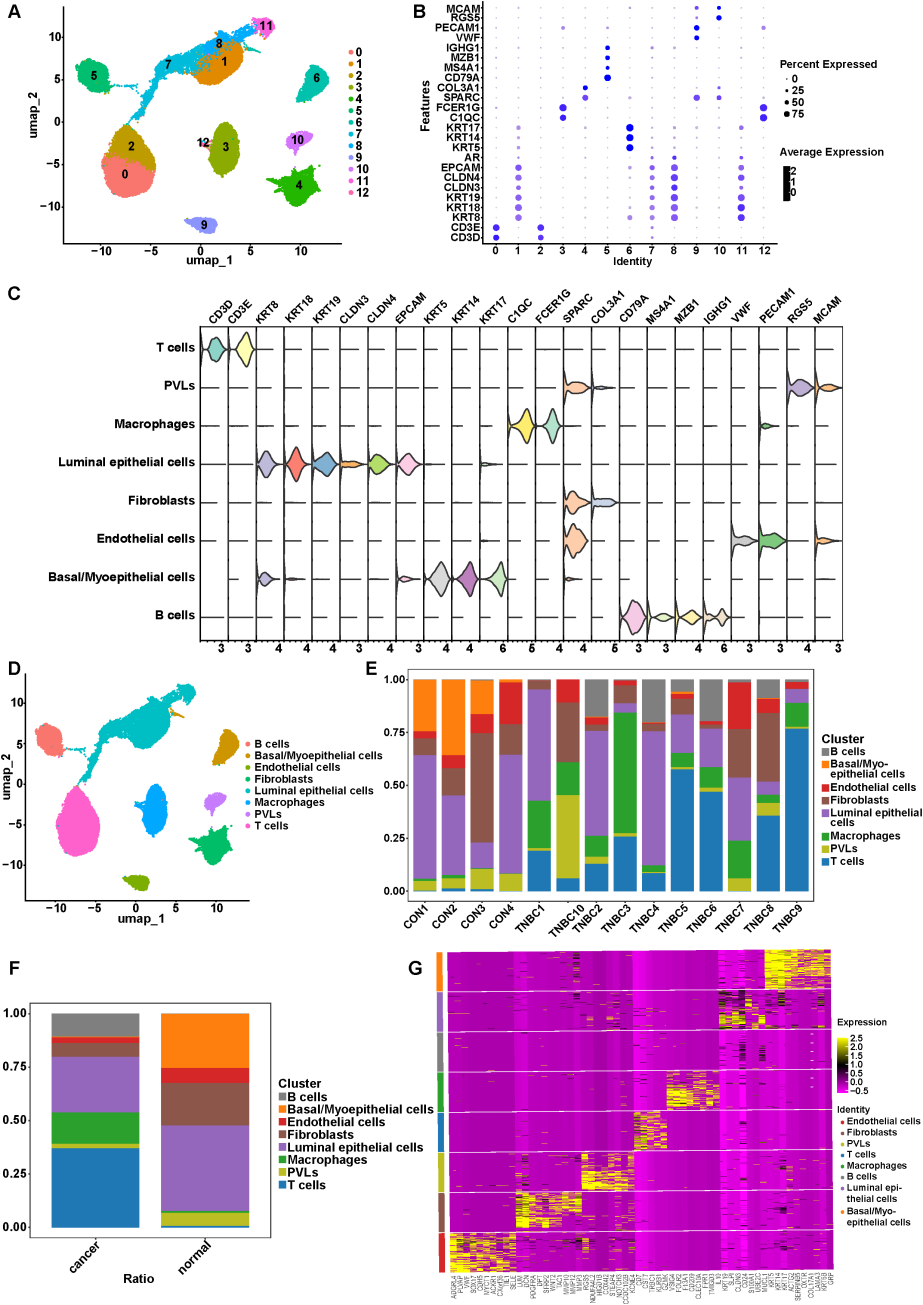
Celltype classification in TNBC. **(A)** UMAP plot of 12 cell clusters. **(B)** Bubble plots of the expression of diagnostic marker genes in each cell cluster. **(C)** Violin plots of the expression of diagnostic marker genes in each celltype. **(D)** UMAP plot of 8 celltypes. **(E)** Cell proportion in each sample. **(F)** Cell proportion in cancer and normal tissue samples. **(G)** Heatmap of the top 10 DEGs in each celltype.

A total of 10 TNBC samples and 4 normal breast tissue samples were included in our scRNA-seq analysis. After stringent QC, 44,364 high-quality cells were retained, including 31,901 cells from tumor tissues and 12,463 cells from normal tissues. Cell clustering and dimensionality reduction were performed using the “Seurat” package. UMAP was employed for dimensionality reduction and visualization, which resolved 12 distinct cell clusters. (**Figure 6A**). These clusters were annotated in accordance with 24 well-established cell-type marker genes. The expression of the marker genes were illustrated using bubble and violin plots. (**Figures 6B, C**). Eight principal cell types were distinguished based on marker gene expression patterns: B cells, luminal epithelial cells, endothelial cells, fibroblasts, macrophages, basal/myoepithelial cells, perivascular-like cells (PVLs), and T cells (**Figure 6D**). Analysis of the cellular composition across samples revealed inter-sample heterogeneity. T cells were the predominant population in TNBC5, TNBC6, TNBC8, and TNBC9, while luminal epithelial cells dominated TNBC1, TNBC2, and TNBC4. PVLs were most abundant in TNBC10, and macrophages represented the largest proportion in TNBC3. In contrast, normal breast tissues were mainly composed of epithelial cells and fibroblasts (**Figure 6E**). When comparing cell type distributions between tumor and normal tissues, immune cells were notably enriched in tumor tissues (**Figure 6F**). Finally, the top 10 DEGs for each cluster were identified and visualized using a heatmap (**Figure 6G**).

### 3.7 The expression of signature genes in single cells

**Figure 7:**
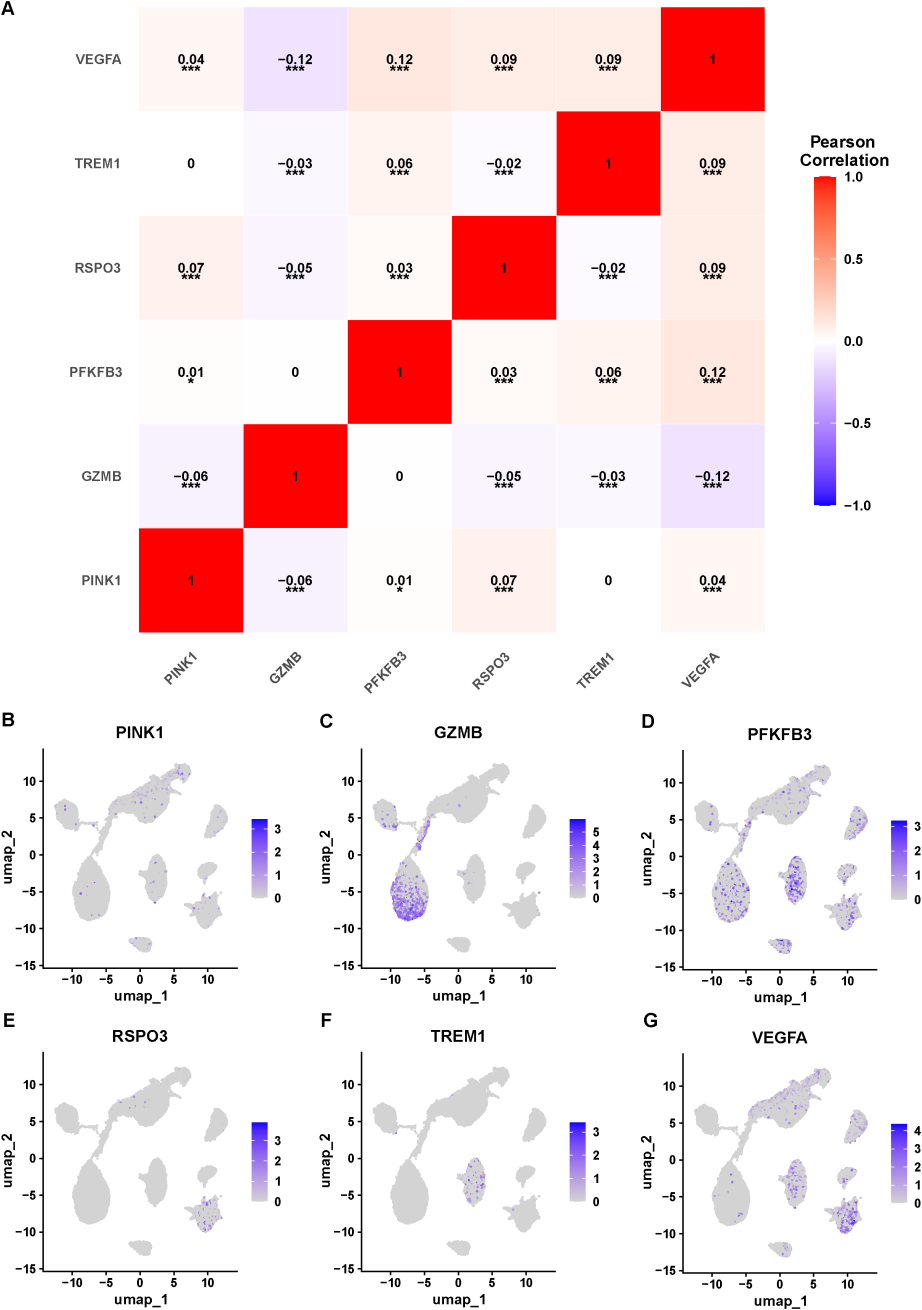
The expression of signature genes in single cells. **(A)** Correlation analysis of 6 signature genes. **(B–G)** UMAP plots of 6 signature genes. *P < 0.05, ***P < 0.001.

To gain deeper insight into the connection between signature genes and specific cell clusters, we analyzed the correlations among 6 signature genes (**Figure 7A**). A modest yet statistically significant positive correlation was observed between PFKFB3 and VEGFA (R = 0.12, P< 0.001), while GZMB showed a negative correlation with VEGFA (R = -0.12, P< 0.001). We then examined the expression of these 6 signature genes across different celltypes (**Figures 7B–G**). PFKFB3 and VEGFA were broadly expressed across multiple cell clusters. TREM1 expression was predominantly enriched in macrophages, whereas GZMB was primarily expressed in T cells. These findings suggest that signature genes may function in a coordinated manner across different cell populations, potentially associated with TNBC progression.

### 3.8 Scoring of signature genes

**Figure 8:**
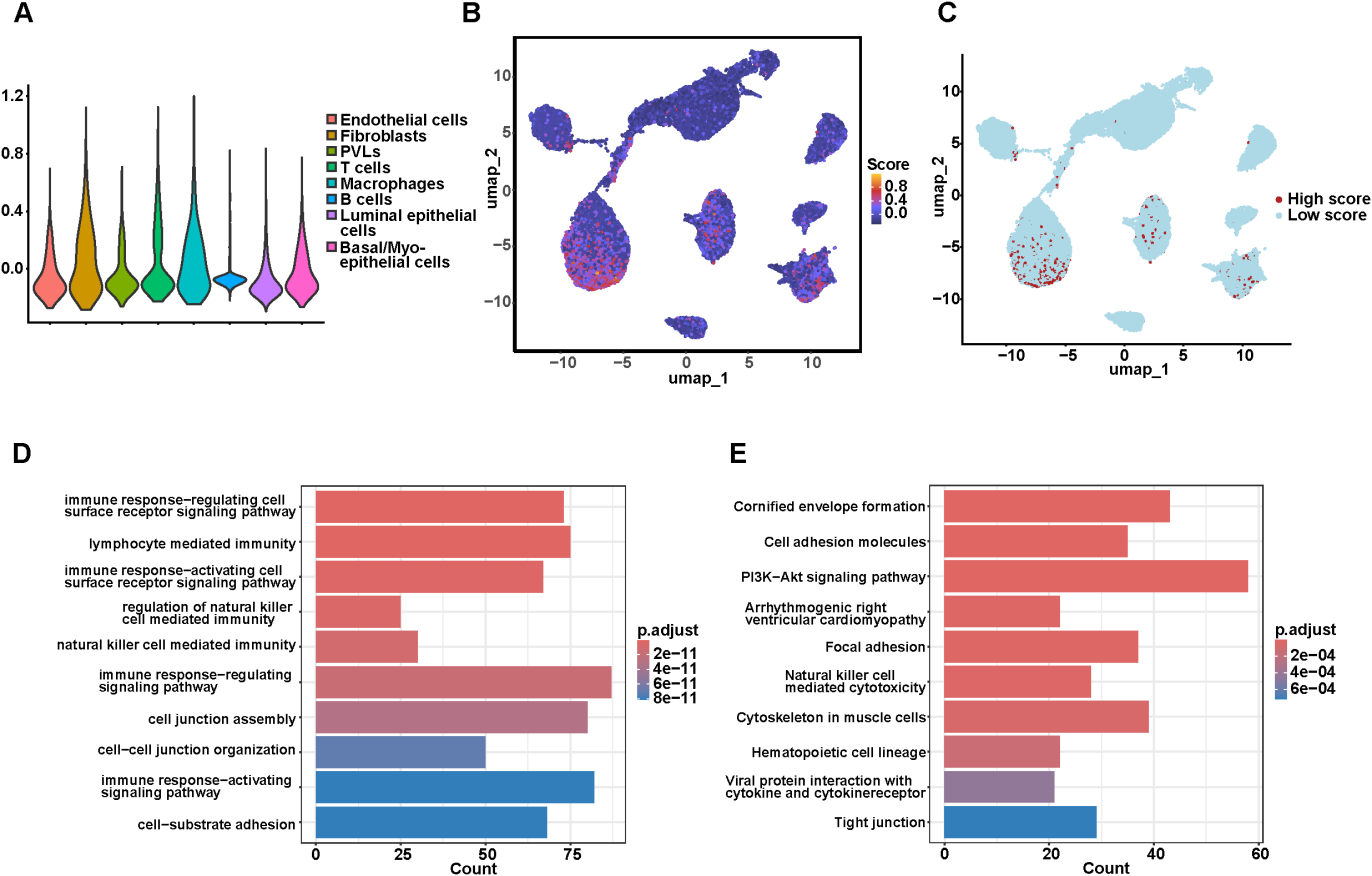
Scoring of 6 signature genes. **(A)** The violin plot of the scoring results. **(B)** The UMAP plot of the scoring results. **(C)** The cell types with high scoring distribution. **(D)** GO analysis of the DEGs in cells with high scores. **(E)** KEGG analysis of the DEGs in cells with high scores.

AddModuleScore from “Seurat” package was employed to calculate signature activity scores for each cell. Generallly, cells with higher expression of signature genes exhibited higher scores. The distribution of the result was visualized using violin plots and UMAP projections (**Figures 8A, B**). The distribution of signature activity score exhibited a right-skewed pattern, with 0.3 identified as the optimal inflection point to distinguish specific pathway activation from background noise (**Supplementary Figure S9**). Importantly, sensitivity analysis across thresholds from 0.2 to 0.4 confirmed the robustness of this cutoff (**Supplementary Table S9**). Therefore, a score of > 0.3 was selected to balance specificity with statistical power for downstream analysis. Using this threshold, a total of 3,848 cells were identified as signature-high. These signature-high cells were predominantly enriched in T cells, macrophages, fibroblasts and luminal epithelial cells (**Figure 8C**). To investigate the functional relevance of these signature-high cells, GO and KEGG enrichment analyses were performed on the DEGs within this population. GO enrichment revealed that signature-high cells were primarily involved in immune-related processes, including regulation of immune response via cell surface receptor signaling, lymphocyte-mediated immunity, activation of immune responses, NK cell-mediated immunity, and cell junction assembly (**Figure 8D**). KEGG analysis indicated that these genes were significantly enriched in pathways including cornified envelope formation, PI3K-Akt signaling, arrhythmogenic right ventricular cardiomyopathy, focal adhesion, NK cell-mediated cytotoxicity, cytoskeletal regulation in muscle cells, and hematopoietic cell lineage (**Figure 8E**).

### 3.9 Subdivision of T cell cluster

**Figure 9:**
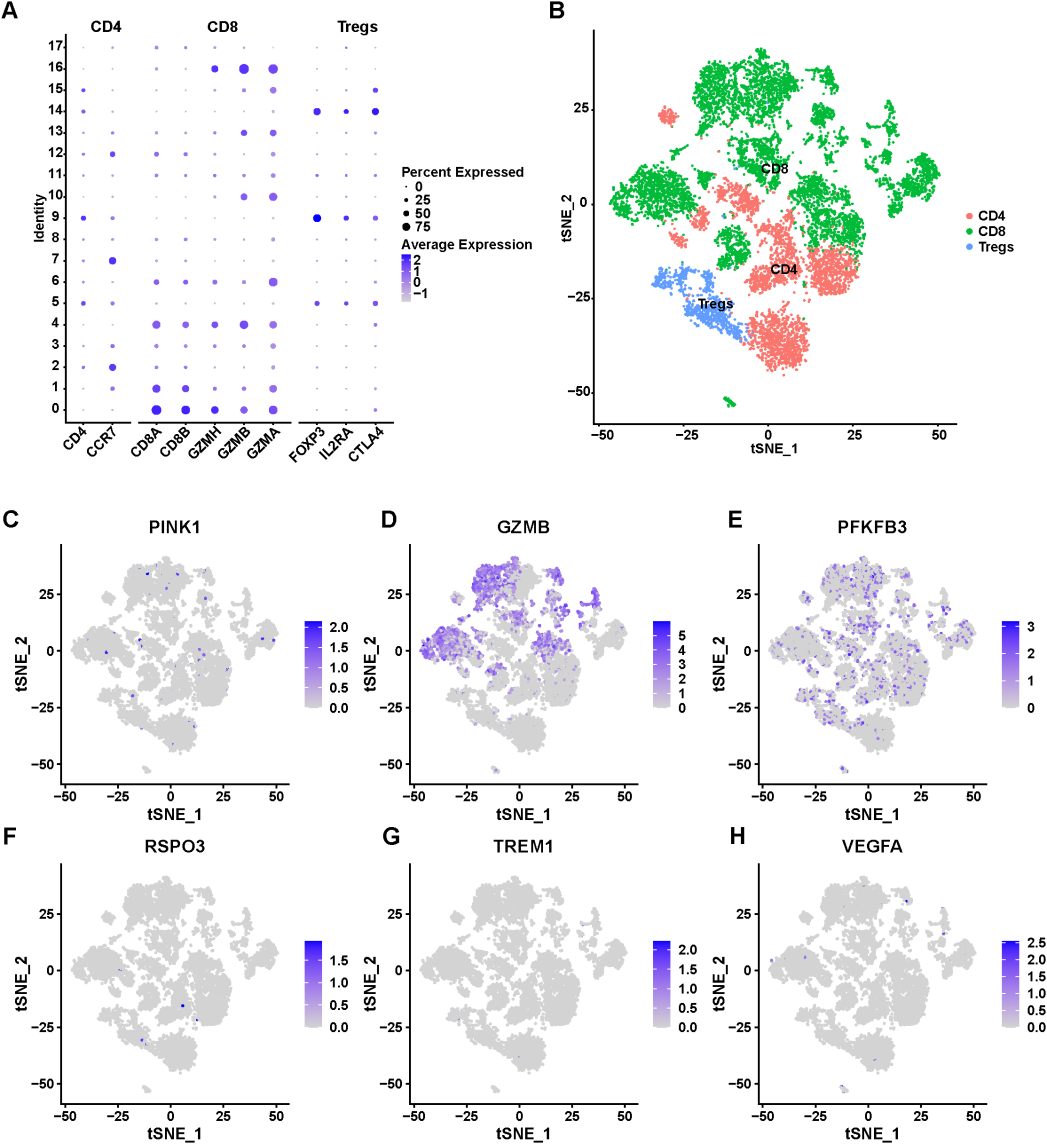
Subdivision of T cell cluster. **(A)** Bubble plots of the expression of cell markers in 3 cell subpopulations. **(B)** t-SNE plots of 3 T cell subpopulations in TNBC. **(C–H)** t-SNE plots of 6 signature genes in 3 cell subpopulations.

The remarkable progress in tumor immunotherapy has brought intermediate T-cell subsets and TIME into greater focus, given their essential contributions to the initiation of anti-tumor immunity (24). Consequently, the expression profiles of the 6 signature genes were assessed in T cells. A total of 11,880 T cells were isolated and further classified into three T cell subpopulations based on canonical immune markers: CD8^+^ T cells (CD8A, CD8B, GZMH, GZMB, GZMA), CD4^+^ T cells (CCR7, CD4), and Tregs (FOXP3, CTLA4, IL2RA) (**Figures 9A, B**). We then examined the expression patterns of 6 signature genes across these T cell subsets (**Figures 9C–H**). PFKFB3 was broadly expressed in all T cell populations. In contrast, PINK1, RSPO3, TREM1, and VEGFA showed minimal or no expression in any subgroup. Notably, GZMB was highly expressed in CD8^+^ T cells, highlighting its potential functional role in this subset.

### 3.10 Pseudotime trajectory analysis

**Figure 10:**
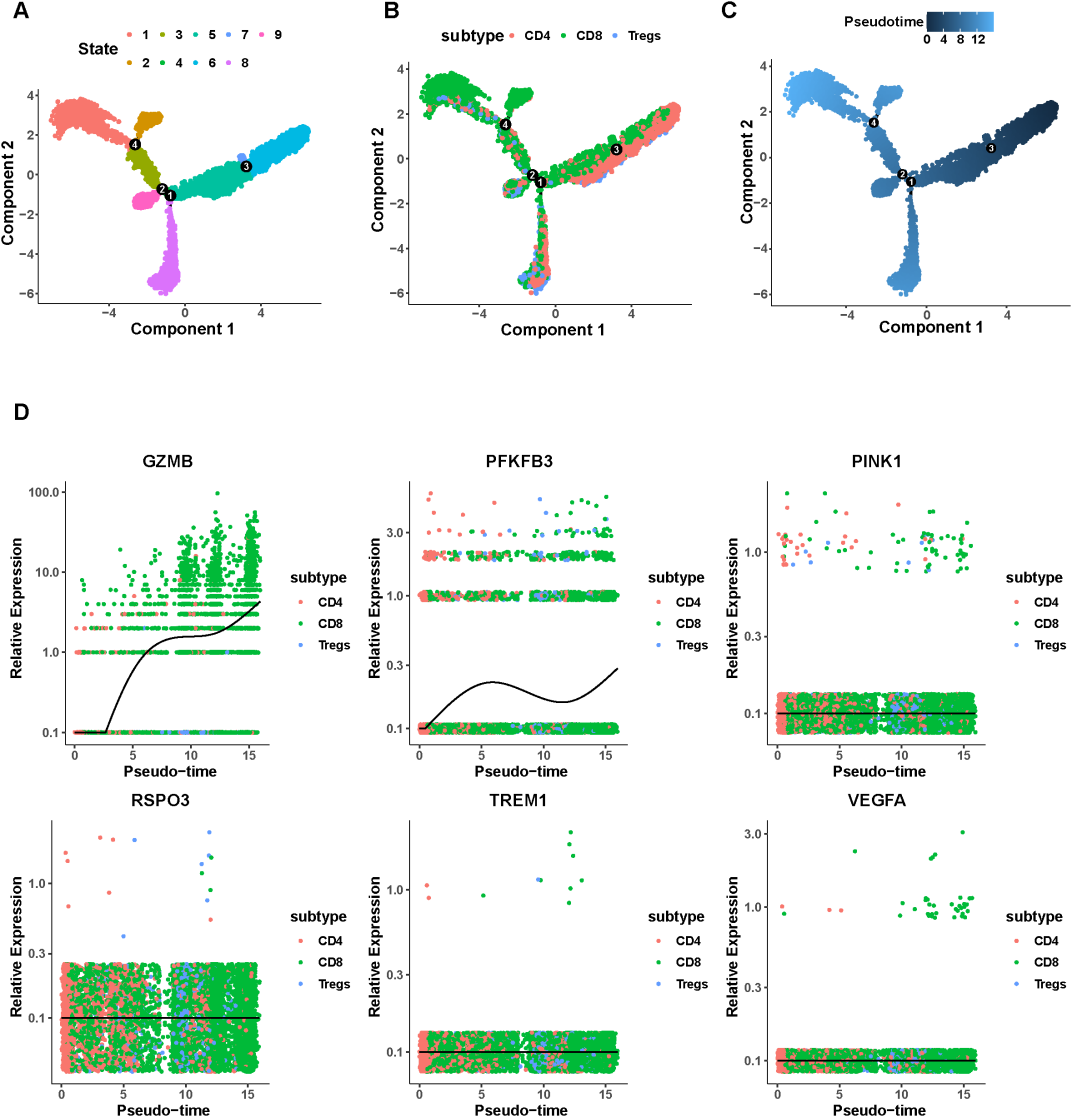
Trajectory analysis of T cells in TNBC. **(A–C)** Transcriptional trajectory of T cells, colored by state **(A)**, cell types **(B)**, and pseudotime **(C)**. **(D)** Relative expression dynamics of 6 signature genes along the pseudotime continuum.

We conducted a pseudotime trajectory analysis using differentially expressed genes between cell clusters to investigate the transcriptional dynamics of all T cells. As shown in **Figure 10A**, nine distinct functional states were identified, each represented by a unique color. Cells located at earlier pseudotime positions (darker blue) likely reflect less activated or naïve-like states, whereas cells at later positions represent more activated or cytotoxic states along a continuous transcriptional spectrum (**Figure 10C**). CD4^+^ T cells were predominantly distributed toward the earlier regions of the pseudotime trajectory, while CD8^+^ T cells and Tregs were more frequently observed in intermediate or later regions. (**Figure 10B**). Crucially, this distribution reflects a transition among distinct T-cell functional states, rather than a direct lineage conversion. The expression patterns of 6 signature genes varied across functional state transitions (**Figure 10D**). Notably, GZMB expression progressively increased along pseudotime, consistent with the transition toward cytotoxic transcriptional states.PFKFB3 exhibited relatively low expression across T-cell states, with a modest increase at intermediate pseudotime stages followed by a gradual decline. In contrast, PINK1, RSPO3, TREM1, and VEGFA showed no consistent expression trends along the pseudotime trajectory.

### 3.11 Cell-cell communication analysis

**Figure 11:**
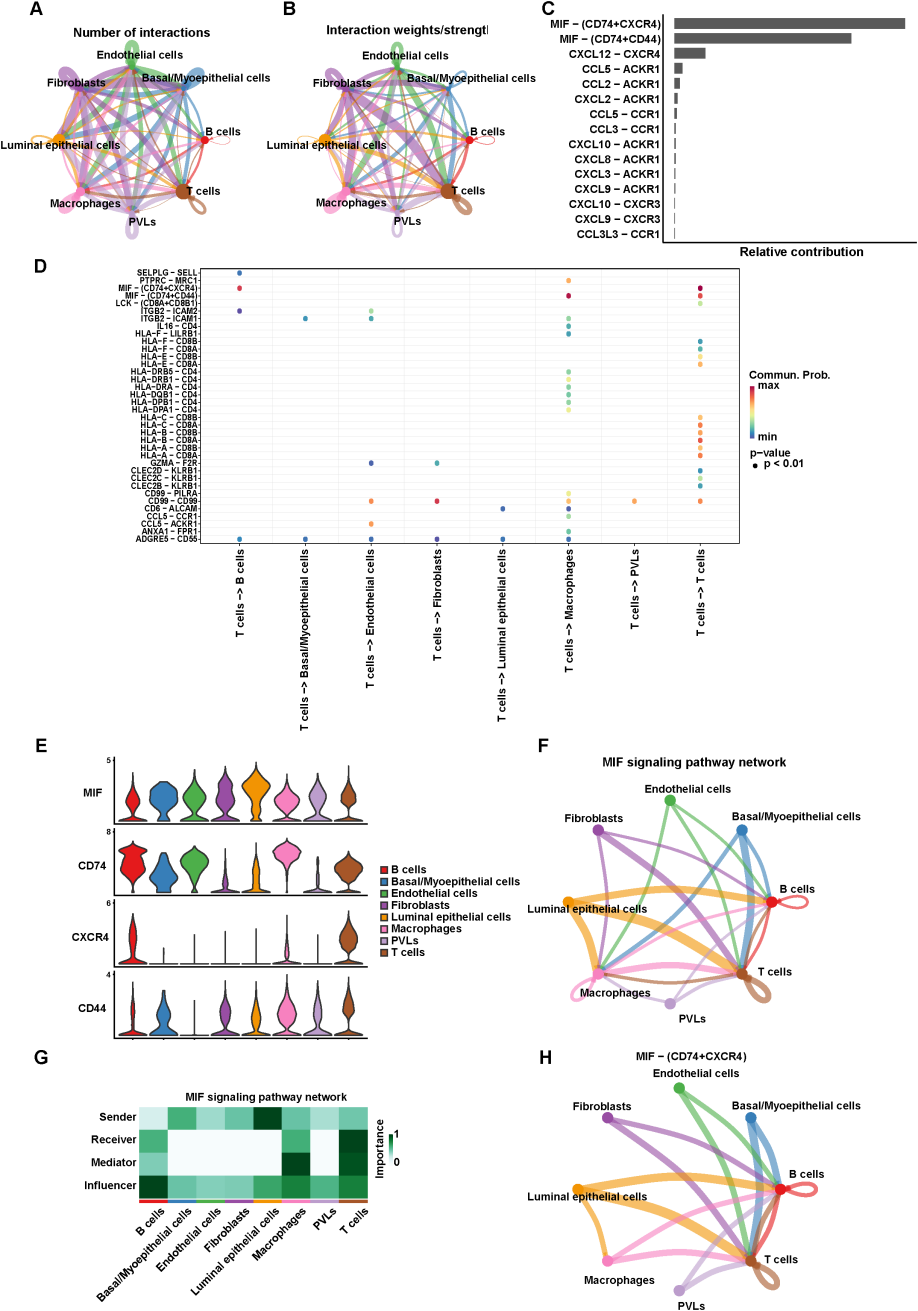
Cell-cell communication analysis. **(A)** The number of interactions in cell-cell communication network. **(B)** The interaction weights/strength in cell-cell communication network. **(C)** The bar chart shows the relative contribution of ligand-receptor. **(D)** Bubble diagram of ligand-receptor pair-mediated interactions between T cells and other cells. **(E)** Violin plot showing the expression of related genes in the MIF signaling pathway. **(F)** Cell-cell communication interaction in MIF signaling pathway. **(G)** The function of single cells in MIF signaling pathway. **(H)** Network visualization of MIF-CD74+CXCR4 axis interactions within cell-cell communication networks.

Intercellular interactions in TNBC were investigated by conducting cell-cell communication analysis across various cell types. With respect to the number of interactions, fibroblasts also showed the most abundant interactions with themselves and other cells (**Figure 11A**). Regarding communication intensity, fibroblasts exhibited the strongest interactions both within their own population and with other cell types (**Figure 11B**). A bar chart was generated to illustrate the receptor-ligand pairs with the highest contributions. The analysis revealed that ligand-receptor-mediated interactions were predominantly enriched in the MIF signaling pathway, particularly involving the pairs MIF-CD74+CXCR4 and MIF-CD74+CD44, with MIF-CD74+CXCR4 contributing the most (**Figure 11C**). Furthermore, a bubble plot was generated to visualize receptor-ligand interactions between T cells and other cells (**Figure 11D**). We next examined the expression profiles of key molecules in MIF signaling pathway across major cell populations (**Figure 11E**). MIF was highly expressed in most cell types. CD74 expression was predominantly enriched in B cells, macrophages, and T cells, whereas CXCR4 was specifically expressed at high levels in B cells and T cells. CD44 displayed a broader distribution, with relatively higher expression in luminal epithelial cells and macrophages. Cell–cell communication network analysis revealed a complex MIF signaling landscape among different cell types (**Figure 11F**). Macrophages served as both major senders and receivers of MIF signals, while T cells engaged in extensive interactions with B cells, macrophages, luminal epithelial cells, and basal/myoepithelial cells. Heatmap analysis of signaling roles (**Figure 11G**) indicated that luminal epithelial cells were the dominant senders, whereas T cells functioned primarily as receivers and influencers in the MIF network. Focusing specifically on MIF-(CD74+CXCR4) ligand-receptor interactions (**Figure 11H**), we observed a more restricted communication pattern, with B cells and T cells forming the principal interaction hubs. Both cell types received signals from multiple populations, suggesting their potential central role in MIF-mediated immune regulation.

### 3.12 Spatial transcriptome analysis in TNBC

**Figure 12:**
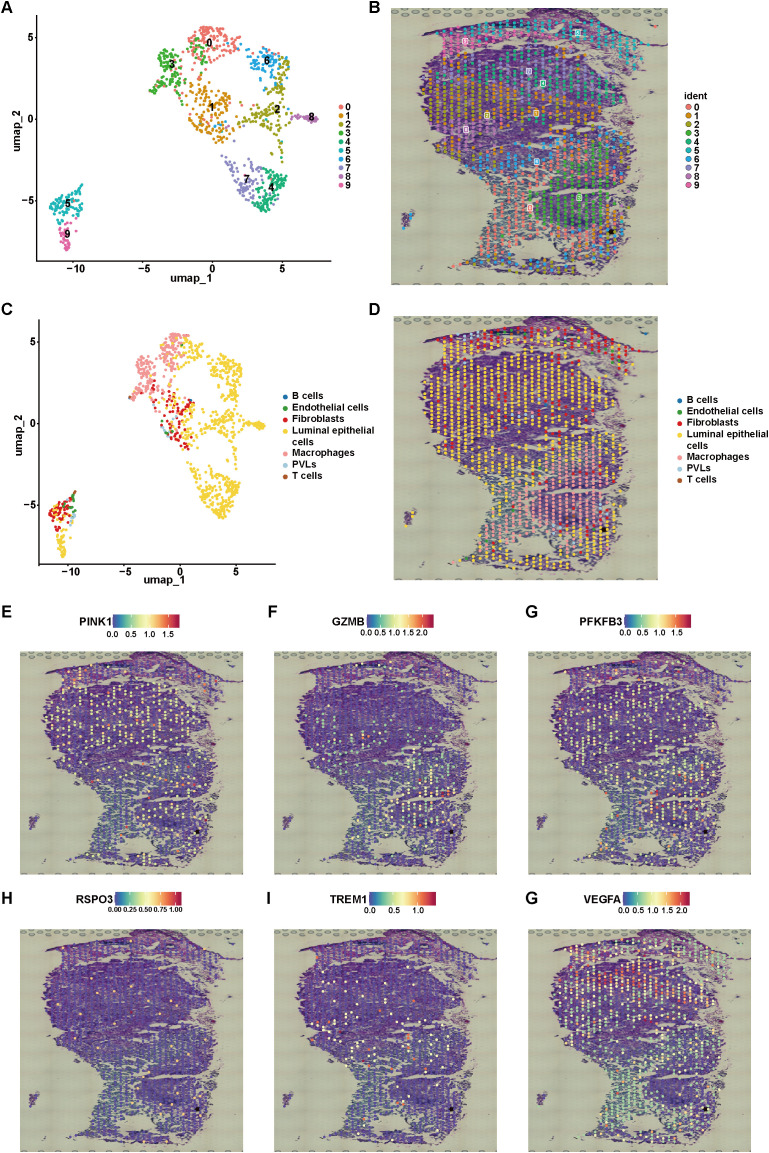
Spatial transcriptome analysis in TNBC. **(A, B)** Dimensionality reduction UMAP plot of ST data and spatial distribution map of each cluster. **(C, D)** Umap plot and spatial distribution of each cell type after annotation. **(E–G)** Spatial distribution of 6 signature genes.

ST enables the investigation of gene expression while preserving the spatial context of the tissue architecture. We analyzed a publicly available TNBC spatial transcriptomics dataset (GSM6433611) using the “Seurat” package. Dimensionality reduction via the UMAP algorithm revealed distinct spatial domains within the tumor tissue (**Figures 12A, B**). Cell-type annotation was performed using the RCTD method, which identified various stromal and immune cell populations. In this specific tissue section, macrophages comprised the largest proportion of immune cells (**Figures 12C, D**). Other immune cell types, such as B cells, NK cells, and T cells, were detected at relatively low frequencies. The spatial expression patterns of 6 signature genes were further examined across the tissue section within this sample (**Figures 12E–G**). These genes exhibited heterogeneous expression patterns, with certain regions showing co-localization with macrophage-enriched areas, suggesting potential associations between these signature genes and the dominant immune compartment. Notably, the expression of some genes was concentrated in stromal regions, while others were distributed across tumor cell-dense regions, indicating potential involvement in distinct microenvironmental niches. These observations provide descriptive evidence of the association of signature genes in distinct microenvironmental niches, warranting further validation in larger spatial cohorts.

### 3.13 IHC validation and prognostic relevance

**Figure 13:**
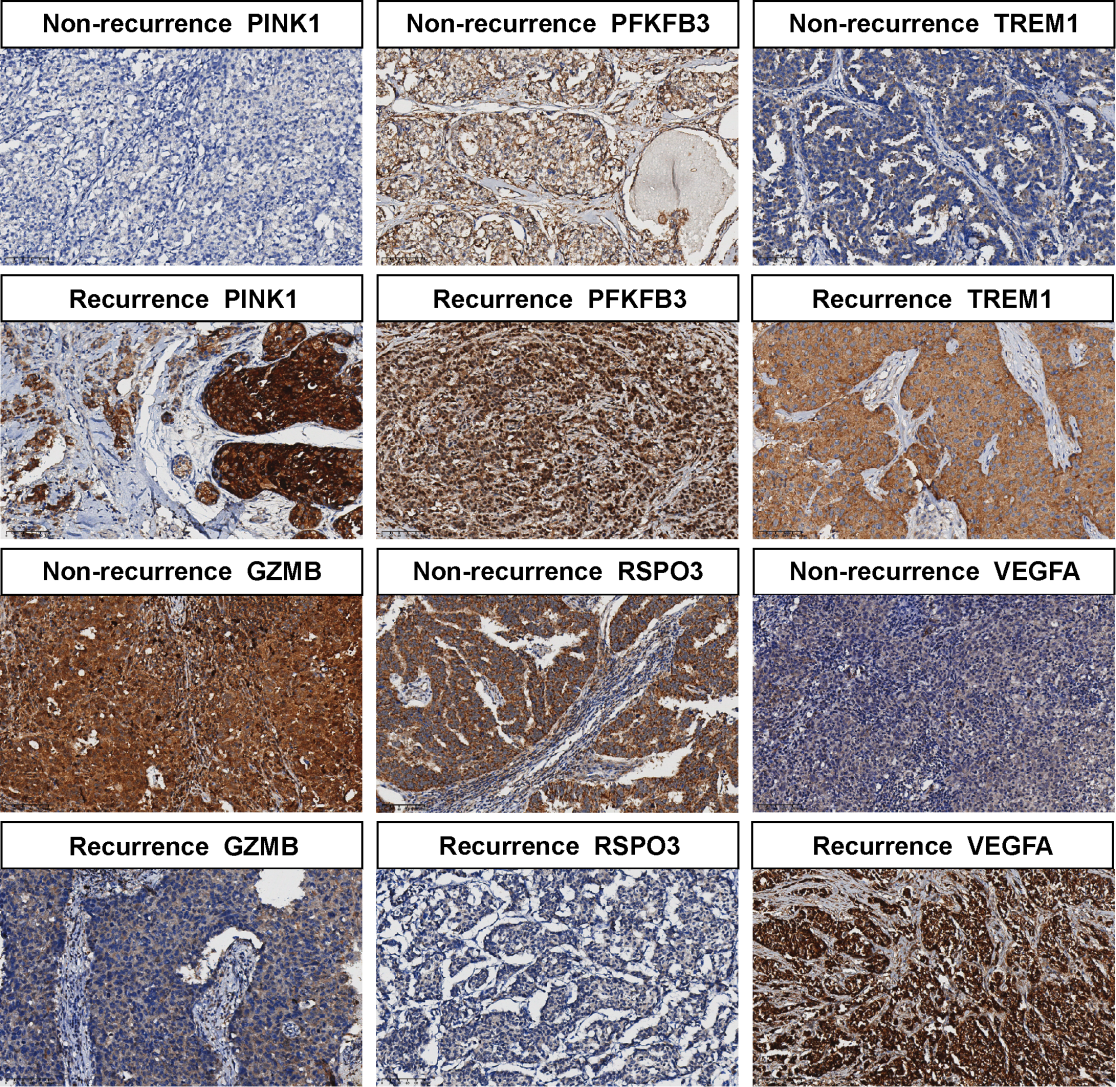
IHC staining of PINK1, GZMB, PFKFB3, RSPO3, TREM1, and VEGFA in the TMAs of patients with TNBC. Scale bar = 100 µm.

For additional confirmation of the predictive ability of signature genes expression in clinical specimens regarding the prognosis of TNBC, a retrospective cohort was assembled of the pathological specimens from 48 TNBC patients. Subsequently, we performed IHC staining on the TMAs of these 48 patients with TNBC (**Figure 13**). Notably, to ensure accurate compartment-specific evaluation, IHC scoring was explicitly restricted to the tumor cell compartment (representative annotated regions are provided in **Supplementary Figure S10**). Furthermore, although the scoring was focused on tumor cells, we acknowledge that for immune-associated markers such as GZMB, the positive signals observed within the tumor cytoplasm likely reflect cytotoxic lymphocyte proximity, interaction, and/or internalization of this protease, rather than endogenous tumor expression. The findings indicated that the expression of PINK1, PFKFB3, TREM1 and VEGFA were markedly higher in recurrent patients than in non-recurrent patients. On the contrary, the expression of GZMB and RSPO3 was visibly higher in non-recurrent patients than in recurrent patients. This is consistent with the associations observed in the transcriptomic analysis.

The chi-square test indicated that tumor recurrence was statistically significantly associated with the expression levels of PINK1, GZMB, PFKFB3, RSPO3, TREM1, and VEGFA, as well as the lymph node metastasis status (**Table 1**). According to the signature score: Risk score = (-0.158360246047182) × GZMB + 0.32235503219597 × PINK1 + 0.111243442274487×PFKFB3 + (-0.0464626296260152) × RSPO3 + 0.0437384204335007 × TREM1 + 0.0364743584502037 × VEGFA. The clinical significance is that a higher score indicates a higher risk, which means a poorer prognosis. Expression levels of GZMB and RSPO3 were negatively linked with the scores. That is, higher expression levels of these two genes were associated with improved prognosis and a reduced likelihood of recurrence. On the contrary, the higher the expression levels of PINK1, PFKFB3, TREM1, and VEGFA, the more likely a recurrence event will occur. A subsequent investigation of associations also confirmed this (**Table 2**).

**Table 1:** Relationship between neoplasm recurrence and clinicopathological features of TNBC.

Characteristic	Cases (n = 48)	Recurrence	Non-recurrence	X^2^	p
Age				0.022	1.000
≤50	27	16	11		
>50	21	12	9		
Histological Grade				4.906	0.115
1	6	1	5		
2	22	14	8		
3	20	13	7		
Lymph nodes metastasis				6.700	0.018*
No metastasis	23	9	14		
Metastasis	25	19	6		
Menstrual Status				0.343	0.770
Premenopause	24	13	11		
Postmenopausal	24	15	9		
Stage				5.066	0.034*
1	1	0	1		
2	34	17	17		
3	13	11	2		
PINK1				6.097	0.023*
Negative	34	16	18		
Positive	14	12	2		
PFKFB3				5.610	0.027*
Negative	15	5	10		
Positive	33	23	10		
TREM1				5.067	0.046*
Negative	35	17	18		
Positive	13	11	2		
GZMB				5.486	0.039*
Negative	24	18	6		
Positive	24	10	14		
RSPO3				6.291	0.019*
Negative	27	20	7		
Positive	21	8	13		
VEGFA				5.976	0.019*
Negative	19	7	12		
Positive	29	21	8		

**Table 2:** Correlation analysis between neoplasm recurrence and clinicopathological features of TNBC.

Characteristic	LN	Stage	PINK1	PFKFB3	TREM1	GZMB	RSPO3	VEGFA
r	0.374	0.349	0.356	0.342	0.325	-0.338	-0.362	0.353
p	0.009*	0.015*	0.013*	0.017*	0.024*	0.019*	0.011*	0.014*

LN, Lymph nodes metastasis.

To assess factors that may predict neoplasm recurrence, both univariate and multivariate regression analyses were conducted, including key clinicopathological factors such as PINK1, GZMB, PFKFB3, RSPO3, TREM1, VEGFA, stage, and lymph nodes metastasis. Univariate analysis indicated that high expressions of GZMB and RSPO3, as well as low expressions of PINK1,PFKFB3, TREM1 and VEGFA, were protective factors linked to reduced tumor recurrence in TNBC. In multivariable logistic regression analyses adjusting for clinicopathological factors (stage and lymph node metastasis), PINK1, GZMB, RSPO3, and VEGFA remained independently associated with tumor recurrence in separate models (**Supplementary Tables S3**–**S8**). PFKFB3 and TREM1 showed borderline significance after adjustment.

**Figure 14:**
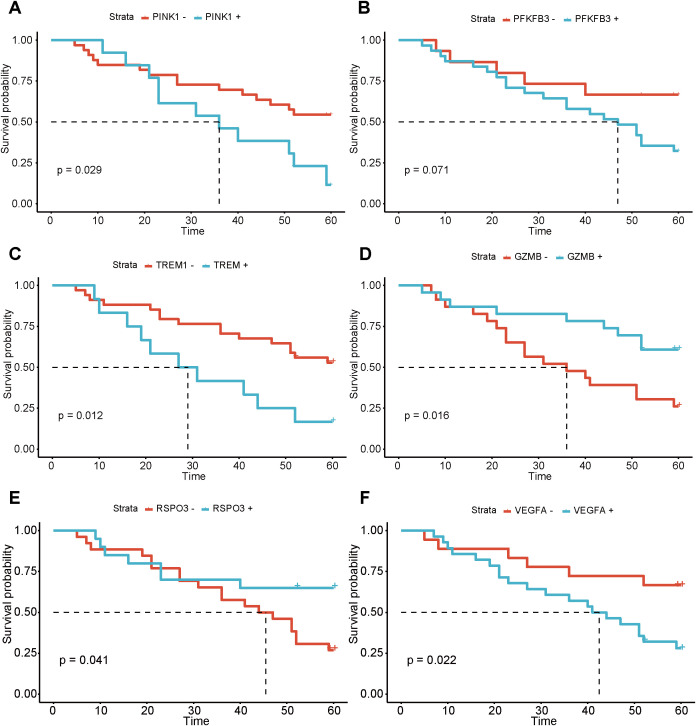
K-M curves of 5-DFS in the clinical TNBC cohort (n = 48). Survival outcomes are stratified by the expression levels of **(A)** PINK1, **(B)** PFKFB3, **(C)** TREM1, **(D)** GZMB, **(E)** RSPO3, and **(F)** VEGFA.

Furthermore, we also plotted a K-M curve based on the expression levels of PINK1, GZMB, PFKFB3, RSPO3, TREM1, and VEGFA in 48 patients with TNBC, as well as the 5-DFS (**Figure 14**). It was observed that patients with high expression of PINK1, TREM1, and VEGFA exhibited significantly poorer 5-DFS compared to those with low expression (p< 0.05). Conversely, individuals exhibiting high levels of GZMB and RSPO3 showed significantly improved 5-DFS (p< 0.05).

## 4 Discussion

BC causes many deaths in women and exhibits distinct epidemiological patterns and heterogeneity. The growing refinement of BC molecular subtypes has improved understanding and enabled more precise therapeutic approaches for the disease (35). TNBC constitutes the most clinically aggressive BC subtype, exhibiting pronounced intratumoral heterogeneity, a high propensity for metastasis, and resistance to chemotherapy, which collectively result in unfavorable clinical outcomes. Although advancements have been made, the mechanisms underlying these aggressive traits are still unclear (36). TNBC stands as the toughest BC subtype to conquer therapeutically (37). The standard treatment strategy consists of surgery in conjunction with chemotherapy and radiotherapy. However, some patients are diagnosed too late to undergo surgery (38).

Pyroptosis is a form of programmed inflammatory cell death characterized by the involvement of the gasdermin (GSDM) family. It significantly influences tumor progression and antitumor immune responses. Pyroptosis acts as a double-edged mechanism, having both pro-tumor and anti-tumor effects (39). Prolonged pyroptosis in cancer cells induced by a hostile TIME can facilitate tumor progression. For instance, GSDME-associated pyroptotic activity has been shown to accelerate the development of colitis-associated colorectal cancer (39, 40). Rapid and robust induction of pyroptosis leads to extensive infiltration of immune cells. This process causes widespread tumor cell death and stimulates antitumor immune responses. These responses help suppress tumor progression (39, 41). Previous studies have indicated that pyroptosis can enhance the anti-tumor effects of certain drugs in TNBC (42, 43). Some studies have already begun to explore new treatment strategies for TNBC by utilizing pyroptosis (44, 45). As mentioned earlier, current studies on PRGs in TNBC mostly rely on a single type of transcriptomic data and lack experimental validation, which limits their reliability and biological interpretability. In this study, we first constructed a PRG signature using bulk transcriptomic data. Then, we integrated scRNA-seq and ST analyses to further explore the heterogeneity of these signature genes across different cellular subpopulations and their association with TIME. This multi-dimensional approach enabled us to delineate the complex cellular interactions and signaling pathways underlying the prognostic relevance of the PRG signature. Moreover, IHC validation using clinical TNBC samples not only confirmed the expression patterns of the signature genes but also included comprehensive prognostic analyses, further verifying their clinical significance. Compared with previous studies, our study gives deeper insights into pyroptosis in TNBC. We validated our findings through experiments and prognostic analysis. This provides stronger clinical evidence and makes our results more convincing.

In this study, we established a PRG signature of TNBC based on PRGs. 6 genes were identified in this prognostic signature: PINK1, GZMB, PFKFB3, RSPO3, TREM1, and VEGFA. PINK1 is a mitochondrial kinase that regulates quality control by recruiting Parkin when mitochondria are damaged. This triggers ubiquitination of outer membrane proteins, leading to proteasomal degradation and selective autophagic clearance of damaged mitochondria (46). GZMB is associated with pyroptosis-related signaling through dual mechanisms: it directly cleaves GSDME at D270 to generate active N-terminal fragments, and indirectly processes GSDME by activating caspase-3. This caspase-independent pathway enables rapid initiation of pyroptosis in target cells (47). PFKFB3 drives glycolytic flux by maintaining high intracellular fructose-2,6-bisphosphate levels through its dominant kinase activity, which allosterically activates PFK-1 to promote glycolysis and support cellular growth (48). RSPO3 amplifies Wnt signaling by binding to LGR receptors. It neutralizes RNF43 and ZNRF3 ubiquitin ligases, which stabilizes Wnt receptors. This enhances β-catenin-mediated transcription, promoting cell proliferation and differentiation. Its dysregulation contributes to tumorigenesis through both cell-autonomous proliferative effects and immunomodulatory functions within TIME (49). RSPO3 has been reported to be linked with pyroptotic activity via activation of the β-catenin-NF-κB axis and NLRP3 inflammasome (33). TREM1 amplifies inflammatory responses through ligand-induced multimerization that triggers downstream signaling pathways, resulting in myeloid cells releasing high levels of pro-inflammatory cytokines and chemokines. This amplification mechanism not only drives pathological inflammation but also promotes tumor progression by modulating TIME and inducing immunosuppression (50).VEGFA promotes tumor angiogenesis via VEGFR2 signaling. It stimulates endothelial cell growth, survival, movement, and vascular permeability. Hypoxia increases VEGFA expression in tumor cells. Its effects are further boosted by interactions with immune cells in the TIME (51). In summary, the 6 signature genes are closely linked to tumor initiation and progression. Furthermore, It is still unknown how these genes jointly coordinate pyroptotic activity associated with the prognosis of TNBC. These findings indicate that these genes may be valuable markers for studying pyroptosis-related landscapes in TNBC.

Relying on the aforementioned signature, a preliminary prognostic evaluation was undertaken. Demonstrably, the risk score generated by the signature achieved strong prognostic performance across distinct clinical endpoints, predicting OS in the training cohort and extending its broader prognostic relevance to DFS in the independent GEO cohort. Subsequent to consensus clustering, TNBC samples were classified into two clusters. Cluster 1 exhibited higher immune scores and more abundant immune cell infiltration compared to cluster 2. This suggests that cluster 1 exhibits an activated immune response state and may be more sensitive to immunotherapy, whereas cluster 2 is likely more prone to immune escape. Next, an analysis of TIME was performed. TIME plays a crucial role in determining tumor progression and therapeutic response in BC. By applying the CIBERSORT algorithm, we systematically evaluated the association between 6 signature genes and immune cell infiltration patterns. Our results exhibited that these genes were distinctly linked with various immune cell subsets, indicating their potential involvement in shaping the immune landscape of TNBC. Among these genes, GZMB and RSPO3 were positively associated with antitumor immune cells. These cells included activated memory CD4^+^ T cells, γδ T cells, M1 macrophages, and plasma cells. The genes were negatively correlated with immunosuppressive populations, such as Tregs and M2 macrophages. These findings suggest that GZMB and RSPO3 may enhance immune activation and promote antitumor immunity. GZMB is a cytotoxic molecule secreted by activated CD8^+^ T cells and NK cells. It contributes to immune-mediated cell death. This is consistent with its association with an immune-activated phenotype (52). RSPO3 enhances antitumor immunity by activating Wnt signaling in NK and CD8^+^ T cells. This activation promotes their infiltration and cytotoxic function. RSPO3 also increases tumor sensitivity to anti-PD-1 therapy (53). In contrast, PINK1, TREM1, and VEGFA showed positive correlations with immunosuppressive immune cells, such as Tregs, M2 macrophages, and monocytes, but negative associations with effector T cells and γδ T cells. These relationships imply that these genes might contribute to immune evasion or tolerance mechanisms within TIME. For instance, by promoting the M2 macrophages, TREM1 contributes to cancer growth and metastatic spread, particularly in the hypoxic TIME (54). In contrast, suppression of TREM1 reprograms these macrophages toward an M1 macrophages by inhibiting PI3K/AKT signaling (55). These observations imply that TREM1 plays a critical role in tumor progression and metastasis. Similarly, VEGF compromises migratory capacity and immune function in mature DCs through the VEGF receptor 2-mediated RhoA-cofilin1 pathway, suggesting that VEGF-induced reduction of mDC motility represents one aspect of tumor immune escape (56). PINK1, a key regulator of mitochondrial homeostasis, may influence immune regulation through its effects on cellular metabolism and oxidative stress. Interestingly, PFKFB3 demonstrated a mixed correlation pattern, being positively associated with both effector cells and regulatory populations. This dual relationship suggests that PFKFB3 may exert context-dependent effects on TIME, possibly by modulating glycolytic metabolism in both immune and tumor cells. Given its pivotal role in cellular energy metabolism, PFKFB3 may act as a metabolic checkpoint that determines immune cell activation and function under different tumor contexts. Collectively, these findings highlight the intricate interplay between signature genes and immune cell infiltration in TNBC. GZMB and RSPO3 appear to foster an immune-activated phenotype, while PINK1, TREM1, and VEGFA are associated with immunosuppressive features that may facilitate tumor immune escape. The heterogeneous immune associations of these genes underscore the intricate and context-dependent characteristics of immune regulation in TNBC and suggest that targeting these pathways could provide novel strategies to enhance antitumor immunity. Immune infiltration analysis indicated that high-risk group has a distinct immune landscape marked by both enhanced immune activation and prominent immunosuppression. Specifically, the increased infiltration of activated NK cells and CD8^+^ T cells suggests an ongoing cytotoxic immune response within TIME. However, this was accompanied by a concurrent enrichment of regulatory Tregs, M0/M2 macrophages, and monocytes, which are well-established immunosuppressive populations that dampen antitumor immunity and facilitate tumor progression. The enrichment of Tregs within tumors dampens antitumor immune responses, with M2 macrophages additionally promoting the formation of an immunosuppressive TIME (57, 58). In contrast, low-risk group exhibited higher proportions of plasma cells, Tfh, and M1 macrophages, cell types typically associated with effective antigen presentation, antibody production, and pro-inflammatory tumoricidal activity. Taken together, these findings imply that despite the presence of cytotoxic immune cells, the high-risk TNBC tumors are dominated by immunosuppressive infiltration, leading to a “paradoxical immune activation state” that promotes immune escape and correlates with poor prognosis.

To obtain deeper biological insights, we integrated single-cell transcriptome data and identified eight distinct cell subpopulations. Gene set scoring revealed that the 6 signature genes were predominantly enriched in T cells, macrophages, fibroblasts, and luminal epithelial cells. Among them, GZMB^+^ CD8^+^ T cells are well known for their crucial role in eliminating malignant cells, a process that relies on the recognition of peptide-MHC class I complexes via their T cell receptor (59). Notably, recent studies have suggested that GZMB not only mediates direct tumor cell killing but can also induce pyroptosis, thereby amplifying anti-tumor immunity through dual mechanisms (34). These findings imply that dysregulated expression of GZMB or other signature genes could disrupt the delicate balance between effective immune surveillance and immune evasion. Hence, therapeutic strategies aimed at restoring CD8^+^ T cell function or enhancing GZMB-driven pyroptosis may hold promise for improving treatment outcomes in TNBC. In parallel, GSDME, a key executor of pyroptosis and a member of the GSDM protein family, has been reported to be predominantly expressed in macrophages (60). This observation agrees with our findings. Signature-high cells were enriched in macrophage populations. This underscores the close connection between macrophages and pyroptotic pathways. Looking ahead, future studies could explore how the interaction between CD8^+^ T cells and macrophages modulates pyroptosis within the TIME. They could also investigate how these mechanisms can be leveraged to design novel immunotherapeutic strategies for TNBC. Considering the pivotal role of CD8^+^ T cells and macrophages in shaping TIME, it is important to clarify how their interactions regulate pyroptosis and contribute to immune modulation in TNBC. Our intercellular communication analysis demonstrated that T cells interacted directly with multiple other cell subtypes. The interactions between T cells and macrophages were particularly strong and functionally relevant. Within these interactions, the MIF pathway stood out as the primary mechanism facilitating intercellular communication. The result highlights MIF’s key role in the TIME. The mechanisms through which interactions between T cells and macrophages mediated by MIF regulate pyroptosis need further investigation.

ST is an innovative approach that allows the examination of gene expression within the preserved spatial context of tissues (23). In our analysis, the 6 signature genes displayed pronounced spatial heterogeneity within TNBC tissues, reflecting the complex and diverse nature of TIME. Interestingly, celltype annotation using RCTD revealed that macrophages emerged as the most abundant population in the immune landscape of the examined tissues, whereas other immune cells such as B cells, NK cells, and T cells were present at much lower levels. This observation suggests that, at least in this TNBC sample, macrophages may play a more dominant role in shaping TIME and potentially modulating tumor behavior. Certain signature genes were spatially co-localized with macrophage-enriched areas. This suggests that these genes might participate in macrophage-related signaling pathways, including pro-inflammatory and anti-inflammatory responses, phagocytosis, and tissue remodeling. The heterogeneous expression of signature genes across stromal and tumor regions further highlights the spatial complexity of TNBC. Such heterogeneity may influence local interactions between tumor cells and the immune compartment, potentially impacting tumor progression and response to therapy. Other immune cell types were poorly represented in these samples. Future studies with larger spatial datasets or multimodal approaches could provide a more complete understanding of how signature genes interact with diverse immune populations in TNBC. In summary, ST analysis reveals the spatial heterogeneity of key genes. It also highlights the dominant role of macrophages in the TNBC TIME. These findings emphasize the importance of considering spatial context when evaluating gene function and therapeutic targets.

The final IHC results also confirmed our findings. IHC was performed in 48 TNBC patients to measure the expression of 6 signature genes: PINK1, GZMB, PFKFB3, RSPO3, TREM1, and VEGFA. The expression of these genes showed significant associations with tumor recurrence and 5-DFS. Chi-square tests demonstrated that recurrence events were correlated not only with these genes but also with lymph node metastasis and tumor stage, highlighting the interplay between molecular features and classical clinicopathological factors. Among the 6 genes, high expression of GZMB and RSPO3 was associated with favorable prognosis, consistent with their negative correlation with the calculated risk score. Importantly, as GZMB is canonically an immune-derived protease, its positive staining observed within the tumor cell compartment likely reflects functional immune delivery, proximity, or internalization from infiltrating cytotoxic lymphocytes, rather than endogenous expression by the tumor cells. Conversely, elevated expression of PINK1, PFKFB3, TREM1, and VEGFA correlated with higher recurrence risk and poorer survival, confirming their role as adverse prognostic indicators. These findings were supported by single- and multivariate regression analyses. The results indicate that the expression of these genes is an independent predictor of TNBC recurrence. The IHC data indicate that these 6 genes capture key aspects of tumor-intrinsic biology. They also capture key aspects of TIME. Their expression patterns can separate TNBC patients into high-risk and low-risk groups for recurrence. This underscores the potential utility of these markers in guiding personalized prognosis and identifying patients who may benefit from targeted therapies, particularly those modulating immune responses or metabolic pathways.

In conclusion, previous findings on pyroptosis-based prognostic markers in TNBC are largely derived from single transcriptomic studies and remain unverified experimentally (61–63). Our study overcomes these limitations by integrating multiple levels of genomic data. We constructed a PRG signature based on bulk sequencing and validated its relevance at the single-cell level, with a particular focus on immune cells such as T cells. Our pseudotime analysis suggests a transcriptional continuum and state transitions across T-cell subpopulations, reflecting shifts in activation and cytotoxic profiles rather than direct lineage conversion. This approach allowed us to uncover dynamic changes in TIME and identify key pathways involved in intercellular communication. ST analysis revealed that signature genes were distributed heterogeneously. The results provide spatial evidence for the important role of TIME in TNBC progression. Our findings were supported by IHC validation using clinical samples. This validation increases the reliability and clinical relevance of the results. Overall, this multi-dimensional analysis not only reinforces the importance of pyroptosis-related mechanisms in TNBC but also offers new insights into potential therapeutic targets within TIME. Our work lays a foundation for future research aimed at developing more effective prognostic and treatment strategies for TNBC patients. Nonetheless, certain limitations remain. The potential causal mechanisms by which these signature genes interact with the pyroptotic machinery remain to be functionally validated. Validation in larger clinical cohorts will be needed. This will enhance the translational applicability of the findings. The results of spatial transcriptomics need to be further validated in a larger sample group of spatial transcriptomes. Furthermore, while our signature demonstrated robust prognostic value across multiple cohorts, we acknowledge the limitation regarding the use of cohort-specific median cut-offs. Because the public transcriptomic datasets were generated across different platforms with varying measurement scales, relative median thresholds were practically employed for patient stratification. However, this relative partitioning lacks direct clinical translatability. In future clinical applications, it will be essential to translate this signature into a standardized clinical assay (e.g., multiplex RT-qPCR or a targeted NanoString panel). Utilizing a large, prospective clinical cohort, an absolute and clinically fixed threshold can then be robustly established using maximally selected rank statistics or time-dependent ROC curve analysis, thereby enabling independent, single-patient risk evaluation. The multivariable analyses regarding protein expression should be interpreted as exploratory and hypothesis-generating, requiring further confirmation in larger populations with multiplex staining to precisely dissect compartment-specific prognostic effects. Despite these challenges, we anticipate that with growing interest and deeper understanding of pyroptosis, these questions will be progressively resolved.

## Data Availability

The original contributions presented in the study are included in the article/**Supplementary Material**. Further inquiries can be directed to the corresponding author.
