# Supplementary material for: Integrating bulk, single-cell, and spatial transcriptomics to identify a novel pyroptosis-related gene signature for predicting prognosis and tumor immune landscape in triple-negative breast cancer
Source: Front Immunol. 2026 Apr 7;17:1743222. doi: 10.3389/fimmu.2026.1743222 (PMC13095739; doi:10.3389/fimmu.2026.1743222)
Supplement: Supplementary file 1 [file DataSheet1.zip › Supplement/Supplementary Figure S6.pdf]

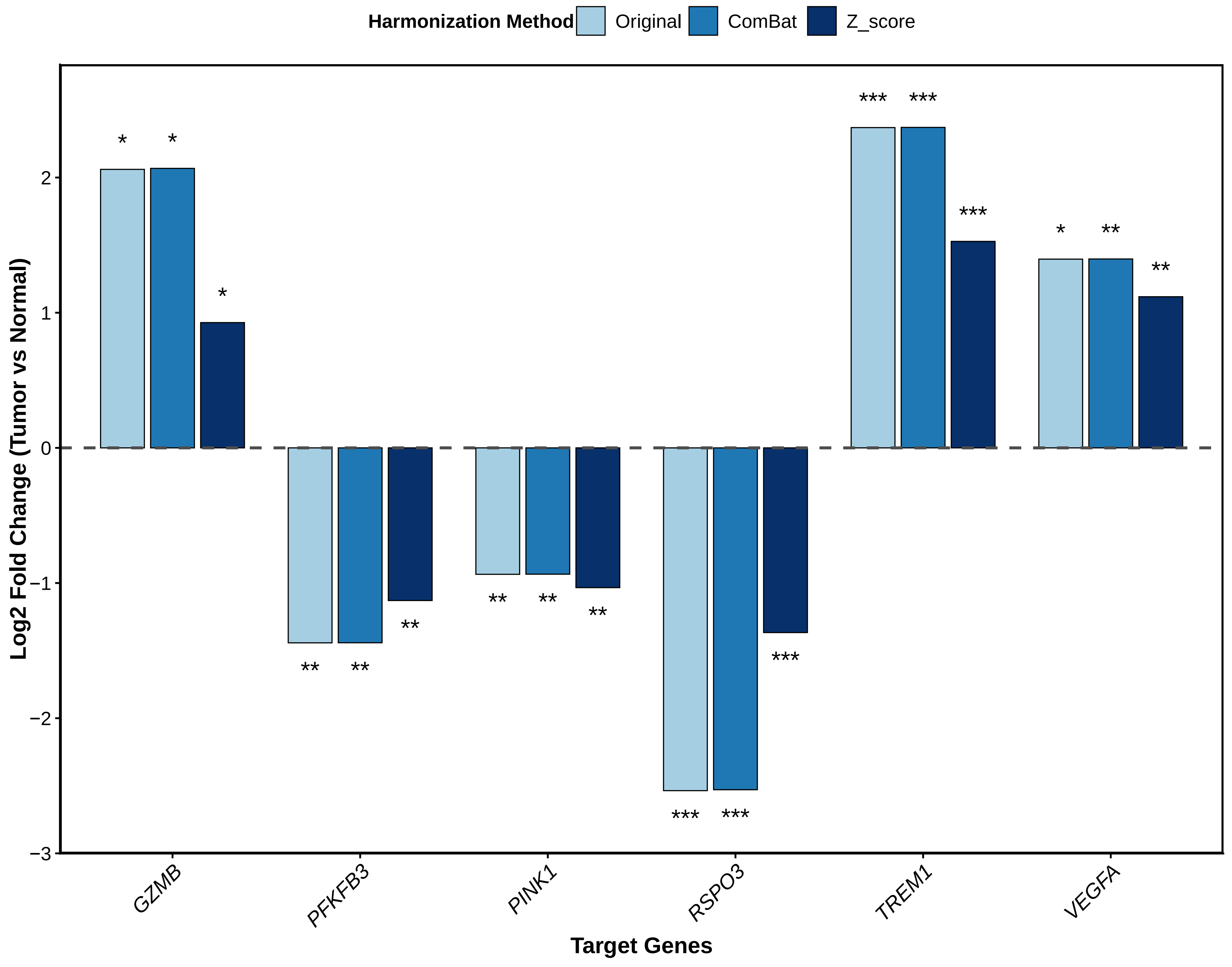
Harmonization Method
Original
ComBat
Z_score
Log2 Fold Change (Tumor vs Normal)
2
1
0
−1
−2
−3
*
*
*
**
**
**
**
**
**
***
***
***
***
***
***
*
**
**
GZMB
PFKFB3
PINK1
RSPO3
TREM1
VEGFA
Target Genes
