# Supplementary material for: Integrating bulk, single-cell, and spatial transcriptomics to identify a novel pyroptosis-related gene signature for predicting prognosis and tumor immune landscape in triple-negative breast cancer
Source: Front Immunol. 2026 Apr 7;17:1743222. doi: 10.3389/fimmu.2026.1743222 (PMC13095739; doi:10.3389/fimmu.2026.1743222)
Supplement: Supplementary file 1 [file DataSheet1.zip › Supplement/Supplementary Tables S3-S8.docx]

**Table S3 Univariate and multivariate logistic regression analysis of factors influencing the tumor recurrence (including PINK1, stage, and lymph nodes metastasis).**

| Variables | Univariate Regression | | | | |  | Multivariate Regression | | | | |
| --- | --- | --- | --- | --- | --- | --- | --- | --- | --- | --- | --- |
|  | β | S.E | Z | *P* | OR (95%CI) |  | β | S.E | Z | *P* | OR (95%CI) |
| PINK1 |  |  |  |  |  |  |  |  |  |  |  |
| Negative |  |  |  |  | 1.00 (Reference) |  |  |  |  |  | 1.00 (Reference) |
| Positive | 1.91 | 0.84 | 2.28 | **0.023** | 6.75 (1.31 ~ 34.85) |  | 1.85 | 0.89 | 2.07 | **0.039** | 6.33 (1.10 ~ 36.44) |
| Stage |  |  |  |  |  |  |  |  |  |  |  |
| 1 |  |  |  |  | 1.00 (Reference) |  |  |  |  |  | 1.00 (Reference) |
| 2 | 16.57 | 2399.54 | 0.01 | 0.994 | 15651360.71 (0.00 ~ Inf) |  | 15.86 | 2399.54 | 0.01 | 0.995 | 7759581.61 (0.00 ~ Inf) |
| 3 | 18.27 | 2399.54 | 0.01 | 0.994 | 86082483.91 (0.00 ~ Inf) |  | 17.30 | 2399.54 | 0.01 | 0.994 | 32682897.32 (0.00 ~ Inf) |
| LN |  |  |  |  |  |  |  |  |  |  |  |
| No metastasis |  |  |  |  | 1.00 (Reference) |  |  |  |  |  | 1.00 (Reference) |
| Metastasis | 1.59 | 0.63 | 2.52 | **0.012** | 4.93 (1.42 ~ 17.06) |  | 0.62 | 0.83 | 0.75 | 0.453 | 1.86 (0.37 ~ 9.38) |
| OR: Odds Ratio, CI: Confidence Interval, LN: Lymph nodes metastasis | | | | | | | | | | | |

**Table S4 Univariate and multivariate logistic regression analysis of factors influencing the tumor recurrence (including PFKFB3, stage, and lymph nodes metastasis).**

| Variables | Univariate Regression | | | | |  | Multivariate Regression | | | | |
| --- | --- | --- | --- | --- | --- | --- | --- | --- | --- | --- | --- |
|  | β | S.E | Z | *P* | OR (95%CI) |  | β | S.E | Z | *P* | OR (95%CI) |
| PFKFB3 |  |  |  |  |  |  |  |  |  |  |  |
| Negative |  |  |  |  | 1.00 (Reference) |  |  |  |  |  | 1.00 (Reference) |
| Positive | 1.53 | 0.67 | 2.29 | **0.022** | 4.60 (1.25 ~ 16.97) |  | 1.48 | 0.79 | 1.86 | 0.063 | 4.38 (0.92 ~ 20.76) |
| Stage |  |  |  |  |  |  |  |  |  |  |  |
| 1 |  |  |  |  | 1.00 (Reference) |  |  |  |  |  | 1.00 (Reference) |
| 2 | 16.57 | 2399.54 | 0.01 | 0.994 | 15651360.71 (0.00 ~ Inf) |  | 16.74 | 2399.54 | 0.01 | 0.994 | 18674474.87 (0.00 ~ Inf) |
| 3 | 18.27 | 2399.54 | 0.01 | 0.994 | 86082483.91 (0.00 ~ Inf) |  | 16.92 | 2399.54 | 0.01 | 0.994 | 22354402.99 (0.00 ~ Inf) |
| LN |  |  |  |  |  |  |  |  |  |  |  |
| No metastasis |  |  |  |  | 1.00 (Reference) |  |  |  |  |  | 1.00 (Reference) |
| Metastasis | 1.59 | 0.63 | 2.52 | **0.012** | 4.93 (1.42 ~ 17.06) |  | 1.35 | 0.83 | 1.62 | 0.105 | 3.85 (0.75 ~ 19.66) |
| OR: Odds Ratio, CI: Confidence Interval, LN: Lymph nodes metastasis | | | | | | | | | | | |

**Table S5 Univariate and multivariate logistic regression analysis of factors influencing the tumor recurrence (including TREM1, stage, and lymph nodes metastasis).**

| Variables | Univariate Regression | | | | |  | Multivariate Regression | | | | |
| --- | --- | --- | --- | --- | --- | --- | --- | --- | --- | --- | --- |
|  | β | S.E | Z | *P* | OR (95%CI) |  | β | S.E | Z | *P* | OR (95%CI) |
| TREM1 |  |  |  |  |  |  |  |  |  |  |  |
| Negative |  |  |  |  | 1.00 (Reference) |  |  |  |  |  | 1.00 (Reference) |
| Positive | 1.76 | 0.84 | 2.10 | **0.036** | 5.82 (1.12 ~ 30.20) |  | 1.69 | 0.89 | 1.90 | 0.058 | 5.41 (0.94 ~ 31.04) |
| Stage |  |  |  |  |  |  |  |  |  |  |  |
| 1 |  |  |  |  | 1.00 (Reference) |  |  |  |  |  | 1.00 (Reference) |
| 2 | 16.57 | 2399.54 | 0.01 | 0.994 | 15651360.71 (0.00 ~ Inf) |  | 15.81 | 2399.54 | 0.01 | 0.995 | 7317692.83 (0.00 ~ Inf) |
| 3 | 18.27 | 2399.54 | 0.01 | 0.994 | 86082483.91 (0.00 ~ Inf) |  | 16.70 | 2399.54 | 0.01 | 0.994 | 17887344.05 (0.00 ~ Inf) |
| LN |  |  |  |  |  |  |  |  |  |  |  |
| No metastasis |  |  |  |  | 1.00 (Reference) |  |  |  |  |  | 1.00 (Reference) |
| Metastasis | 1.59 | 0.63 | 2.52 | **0.012** | 4.93 (1.42 ~ 17.06) |  | 1.14 | 0.79 | 1.44 | 0.150 | 3.12 (0.66 ~ 14.66) |
| OR: Odds Ratio, CI: Confidence Interval, LN: Lymph nodes metastasis | | | | | | | | | | | |

**Table S6 Univariate and multivariate logistic regression analysis of factors influencing the tumor recurrence (including GZMB, stage, and lymph nodes metastasis).**

| Variables | Univariate Regression | | | | |  | Multivariate Regression | | | | |
| --- | --- | --- | --- | --- | --- | --- | --- | --- | --- | --- | --- |
|  | β | S.E | Z | *P* | OR (95%CI) |  | β | S.E | Z | *P* | OR (95%CI) |
| GZMB |  |  |  |  |  |  |  |  |  |  |  |
| Negative |  |  |  |  | 1.00 (Reference) |  |  |  |  |  | 1.00 (Reference) |
| Positive | -1.44 | 0.63 | -2.29 | **0.022** | 0.24 (0.07 ~ 0.81) |  | -2.01 | 0.78 | -2.57 | **0.010** | 0.13 (0.03 ~ 0.62) |
| Stage |  |  |  |  |  |  |  |  |  |  |  |
| 1 |  |  |  |  | 1.00 (Reference) |  |  |  |  |  | 1.00 (Reference) |
| 2 | 16.57 | 2399.54 | 0.01 | 0.994 | 15651360.71 (0.00 ~ Inf) |  | 14.99 | 2399.54 | 0.01 | 0.995 | 3240340.94 (0.00 ~ Inf) |
| 3 | 18.27 | 2399.54 | 0.01 | 0.994 | 86082483.91 (0.00 ~ Inf) |  | 16.55 | 2399.55 | 0.01 | 0.994 | 15338313.35 (0.00 ~ Inf) |
| LN |  |  |  |  |  |  |  |  |  |  |  |
| No metastasis |  |  |  |  | 1.00 (Reference) |  |  |  |  |  | 1.00 (Reference) |
| Metastasis | 1.59 | 0.63 | 2.52 | **0.012** | 4.93 (1.42 ~ 17.06) |  | 1.25 | 0.86 | 1.46 | 0.145 | 3.49 (0.65 ~ 18.70) |
| OR: Odds Ratio, CI: Confidence Interval, LN: Lymph nodes metastasis | | | | | | | | | | | |

**Table S7 Univariate and multivariate logistic regression analysis of factors influencing the tumor recurrence (including RSPO3, stage, and lymph nodes metastasis).**

| Variables | Univariate Regression | | | | |  | Multivariate Regression | | | | |
| --- | --- | --- | --- | --- | --- | --- | --- | --- | --- | --- | --- |
|  | β | S.E | Z | *P* | OR (95%CI) |  | β | S.E | Z | *P* | OR (95%CI) |
| RSPO3 |  |  |  |  |  |  |  |  |  |  |  |
| Negative |  |  |  |  | 1.00 (Reference) |  |  |  |  |  | 1.00 (Reference) |
| Positive | -1.54 | 0.63 | -2.44 | **0.015** | 0.22 (0.06 ~ 0.74) |  | -1.40 | 0.68 | -2.05 | **0.040** | 0.25 (0.07 ~ 0.94) |
| Stage |  |  |  |  |  |  |  |  |  |  |  |
| 1 |  |  |  |  | 1.00 (Reference) |  |  |  |  |  | 1.00 (Reference) |
| 2 | 16.57 | 2399.54 | 0.01 | 0.994 | 15651360.71 (0.00 ~ Inf) |  | 15.45 | 2399.54 | 0.01 | 0.995 | 5152374.21 (0.00 ~ Inf) |
| 3 | 18.27 | 2399.54 | 0.01 | 0.994 | 86082483.91 (0.00 ~ Inf) |  | 16.39 | 2399.55 | 0.01 | 0.995 | 13103935.45 (0.00 ~ Inf) |
| LN |  |  |  |  |  |  |  |  |  |  |  |
| No metastasis |  |  |  |  | 1.00 (Reference) |  |  |  |  |  | 1.00 (Reference) |
| Metastasis | 1.59 | 0.63 | 2.52 | **0.012** | 4.93 (1.42 ~ 17.06) |  | 1.06 | 0.80 | 1.34 | 0.180 | 2.90 (0.61 ~ 13.78) |
| OR: Odds Ratio, CI: Confidence Interval, LN: Lymph nodes metastasis | | | | | | | | | | | |

**Table S8 Univariate and multivariate logistic regression analysis of factors influencing the tumor recurrence (including VEGFA, stage, and lymph nodes metastasis).**

| Variables | Univariate Regression | | | | |  | Multivariate Regression | | | | |
| --- | --- | --- | --- | --- | --- | --- | --- | --- | --- | --- | --- |
|  | β | S.E | Z | *P* | OR (95%CI) |  | β | S.E | Z | *P* | OR (95%CI) |
| VEGFA |  |  |  |  |  |  |  |  |  |  |  |
| Negative |  |  |  |  | 1.00 (Reference) |  |  |  |  |  | 1.00 (Reference) |
| Positive | 1.50 | 0.63 | 2.38 | **0.017** | 4.50 (1.31 ~ 15.52) |  | 1.40 | 0.69 | 2.03 | **0.043** | 4.06 (1.05 ~ 15.71) |
| Stage |  |  |  |  |  |  |  |  |  |  |  |
| 1 |  |  |  |  | 1.00 (Reference) |  |  |  |  |  | 1.00 (Reference) |
| 2 | 16.57 | 2399.54 | 0.01 | 0.994 | 15651360.71 (0.00 ~ Inf) |  | 15.38 | 2399.54 | 0.01 | 0.995 | 4800430.53 (0.00 ~ Inf) |
| 3 | 18.27 | 2399.54 | 0.01 | 0.994 | 86082483.91 (0.00 ~ Inf) |  | 16.45 | 2399.55 | 0.01 | 0.995 | 13907678.20 (0.00 ~ Inf) |
| LN |  |  |  |  |  |  |  |  |  |  |  |
| No metastasis |  |  |  |  | 1.00 (Reference) |  |  |  |  |  | 1.00 (Reference) |
| Metastasis | 1.59 | 0.63 | 2.52 | **0.012** | 4.93 (1.42 ~ 17.06) |  | 1.00 | 0.79 | 1.27 | 0.206 | 2.73 (0.58 ~ 12.88) |
| OR: Odds Ratio, CI: Confidence Interval, LN: Lymph nodes metastasis | | | | | | | | | | | |
