# Supplementary material for: Integrating bulk, single-cell, and spatial transcriptomics to identify a novel pyroptosis-related gene signature for predicting prognosis and tumor immune landscape in triple-negative breast cancer
Source: Front Immunol. 2026 Apr 7;17:1743222. doi: 10.3389/fimmu.2026.1743222 (PMC13095739; doi:10.3389/fimmu.2026.1743222)
Supplement: Supplementary file 1 [file DataSheet1.zip › Supplement/Supplementary Tables S9.docx]

| Threshold | Cell Count | Percentage (%) | Dominant Enriched Cluster |
| --- | --- | --- | --- |
| > 0.1 | 10207 | 23.01 | (Background noise included) |
| > 0.2 | 6530 | 14.72 | T cells (41.5%) |
| > 0.3 | 3848 | 8.67 | T cells (48.9%) |
| > 0.4 | 2291 | 5.16 | T cells (52.1%) |
| > 0.5 | 1247 | 2.81 | (Insufficient statistical power) |

Supplementary Table S9. Sensitivity Analysis of Threshold Selection
