# Supplementary figures and images for: Integrating bulk, single-cell, and spatial transcriptomics to identify a novel pyroptosis-related gene signature for predicting prognosis and tumor immune landscape in triple-negative breast cancer
Source: Front Immunol. 2026 Apr 7;17:1743222. doi: 10.3389/fimmu.2026.1743222 (PMC13095739; doi:10.3389/fimmu.2026.1743222)

PCA before vs after batch correction

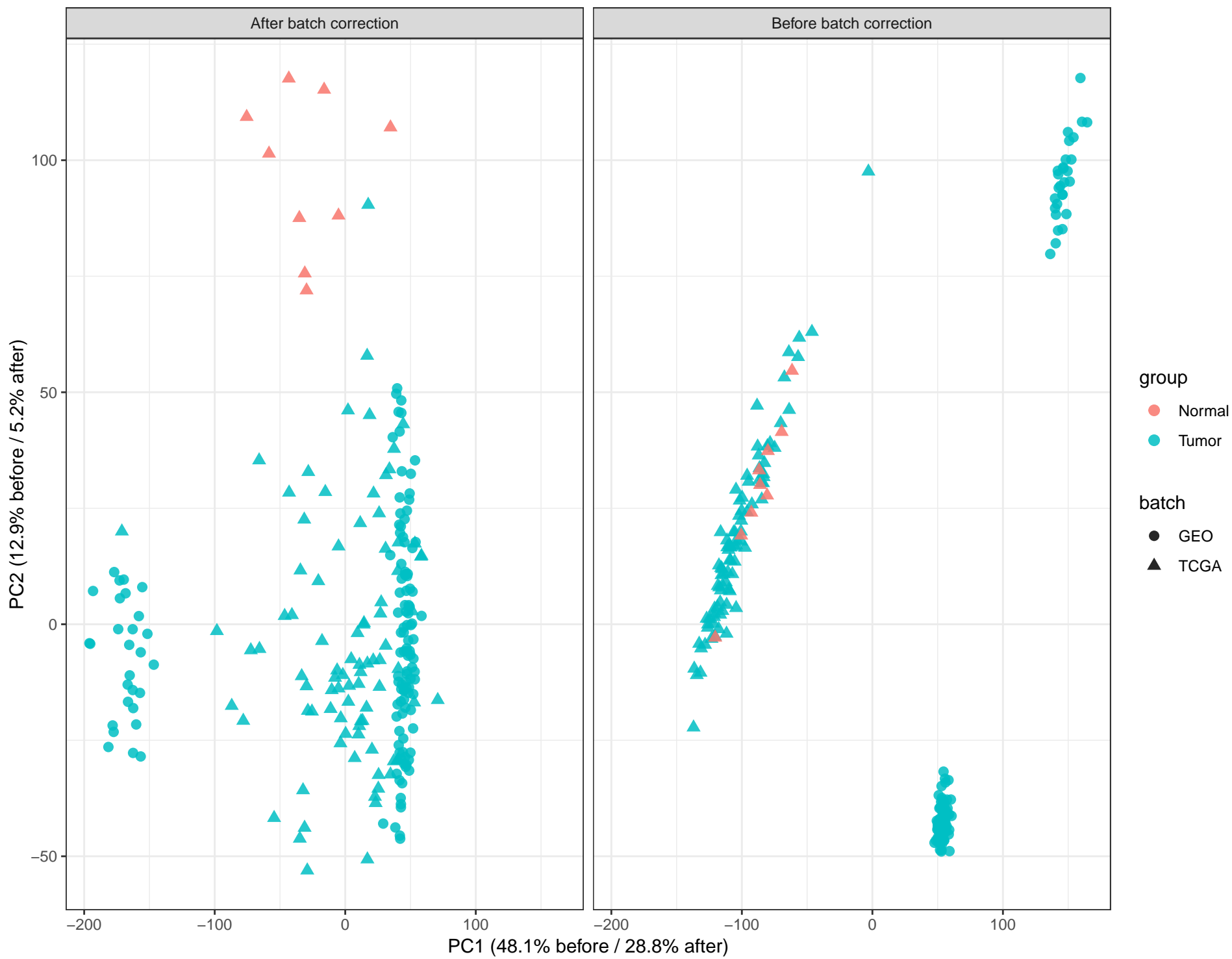

Supplement: Supplementary file 1 [file DataSheet1.zip › Supplement/Supplementary Figure S1.pdf]

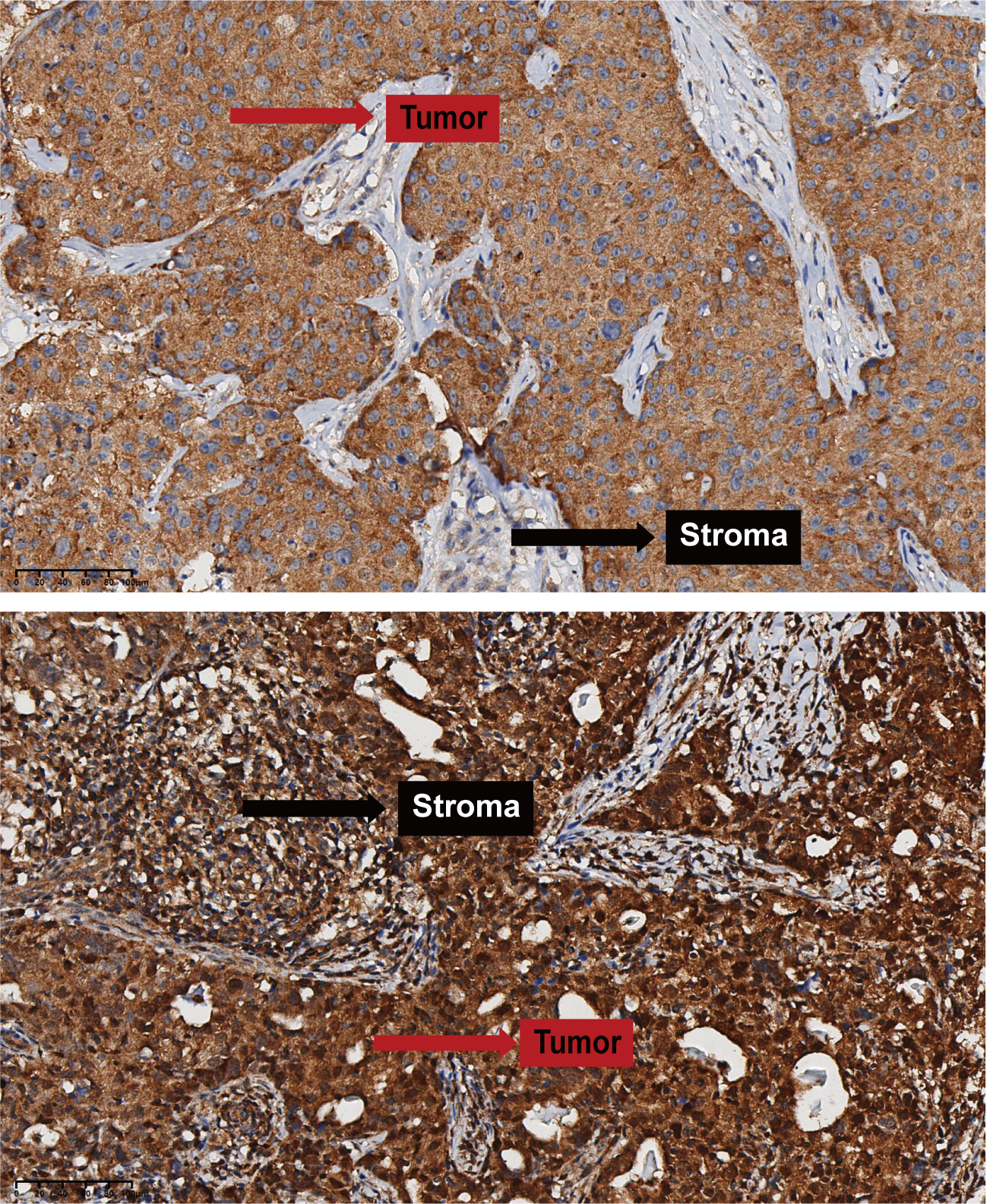

Supplement: Supplementary file 1 [file DataSheet1.zip › Supplement/Supplementary Figure S10.tif]

**Spearman rho = -0.415**

**p = 2.76e-09**

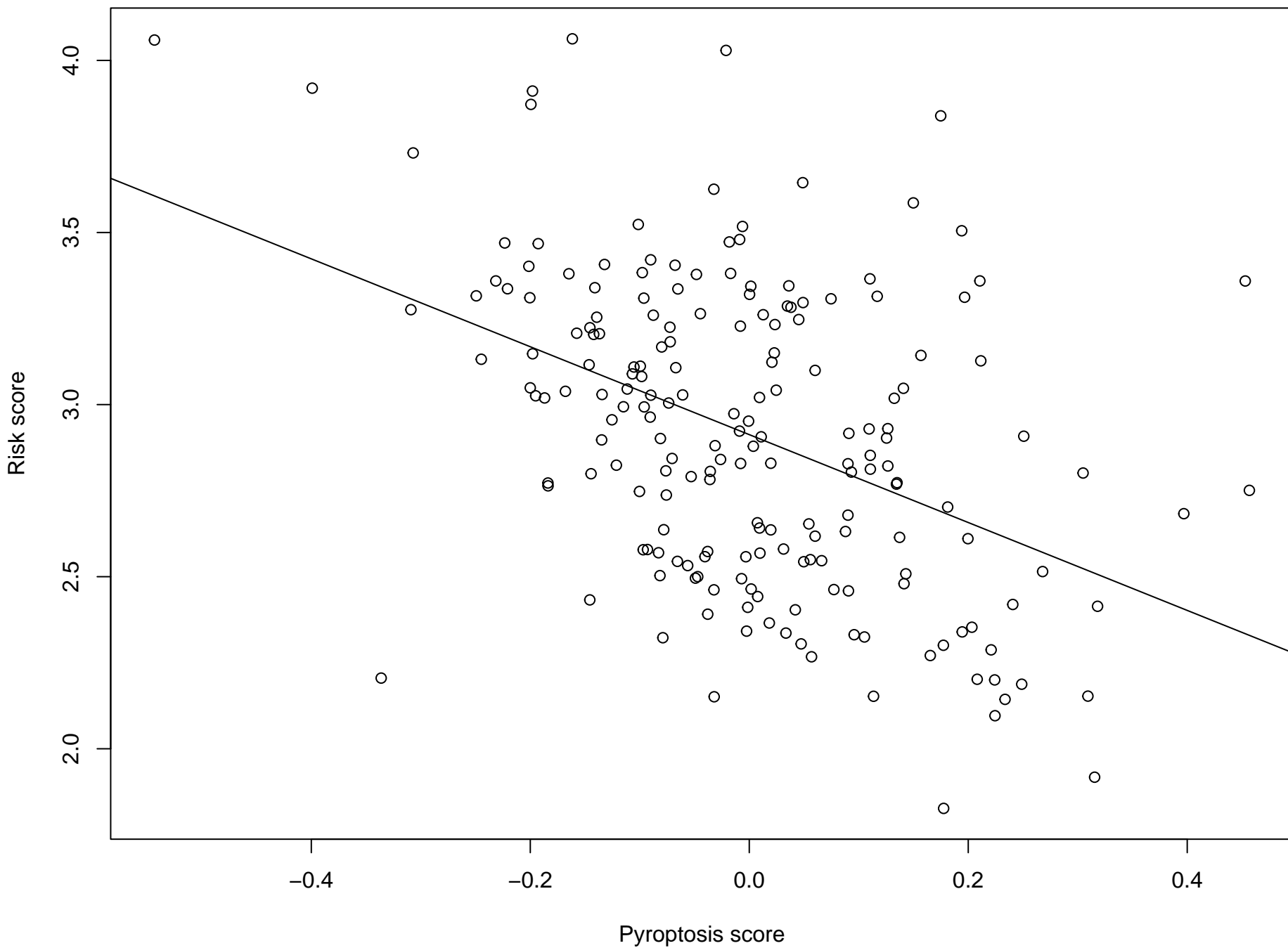

Supplement: Supplementary file 1 [file DataSheet1.zip › Supplement/Supplementary Figure S2.pdf]

## Sensitivity Analysis

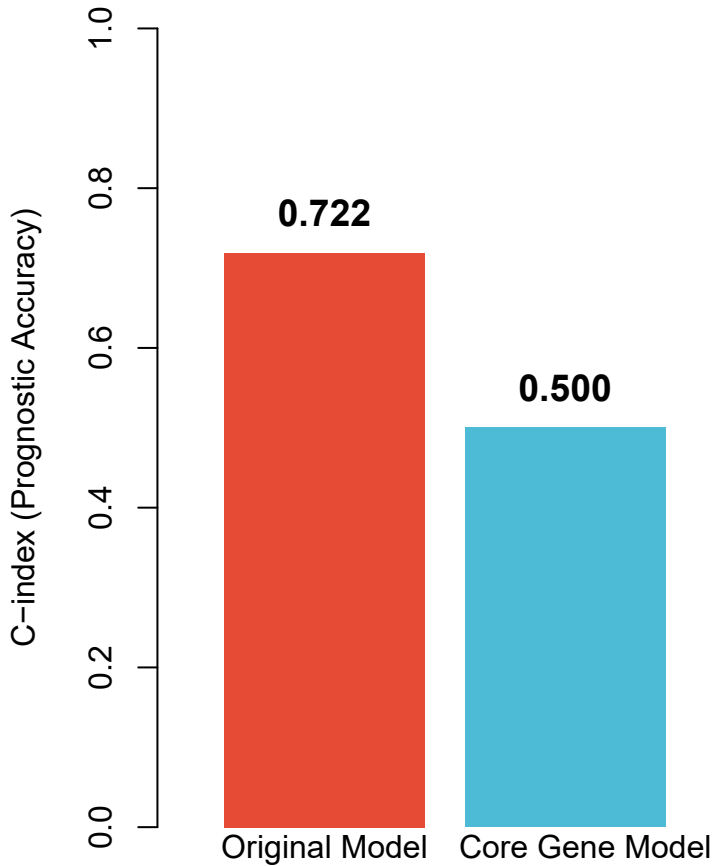

Supplement: Supplementary file 1 [file DataSheet1.zip › Supplement/Supplementary Figure S3.pdf]

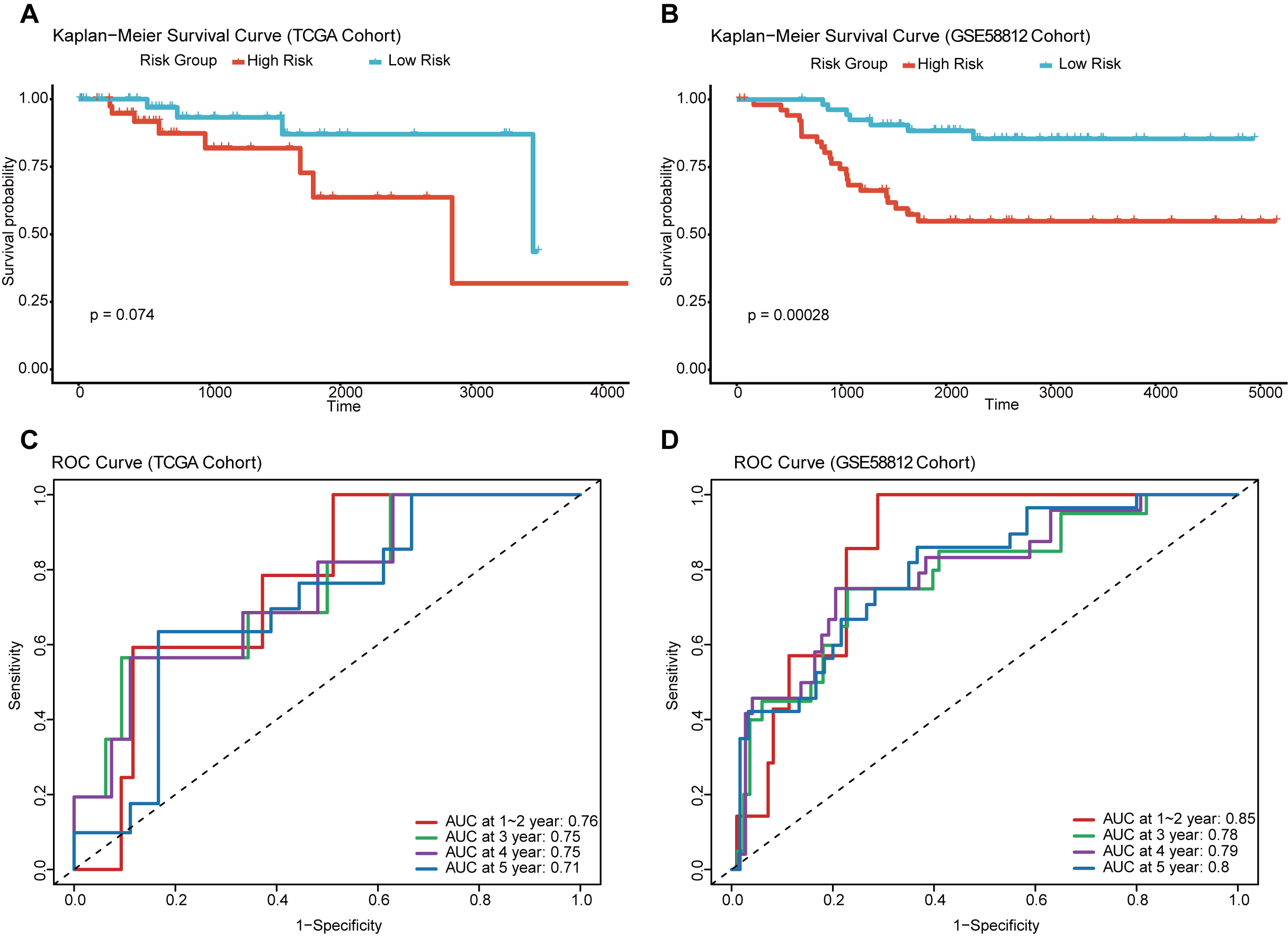

Supplement: Supplementary file 1 [file DataSheet1.zip › Supplement/Supplementary Figure S4.tif]

Cohort

HR (per SD)

95% CI

P Value

Training Cohort

1.926

1.445–2.567

0.0000

GEO Cohort

2.780

1.993–3.880

0.0000

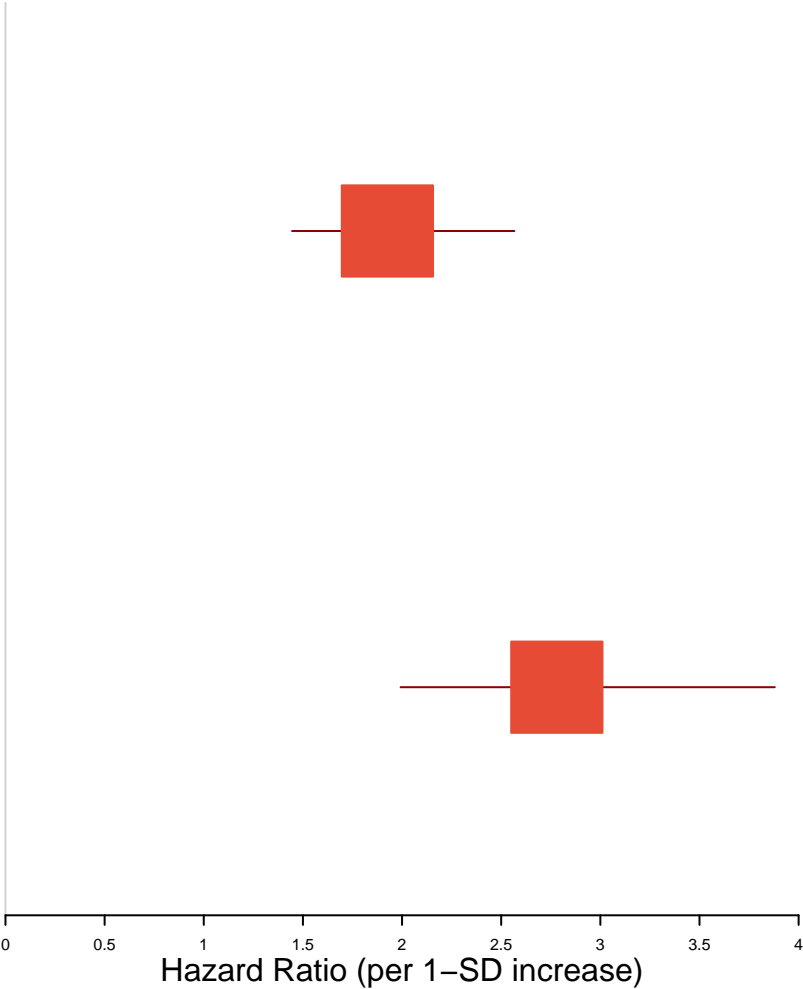

Supplement: Supplementary file 1 [file DataSheet1.zip › Supplement/Supplementary Figure S5.pdf]

# Multivariate Cox Analysis

**RiskScore**

(N=193)

7.2  
(2.9 - 18)

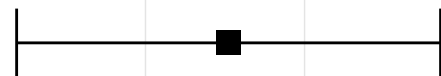

<0.001 \*

**ImmuneScore**

(N=193)

1.0  
(1.0 - 1)

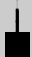

0.138

# Events: 41; Global p-value (Log-Rank): 2.8585e-05

Supplement: Supplementary file 1 [file DataSheet1.zip › Supplement/Supplementary Figure S7.pdf]

# Incremental Multivariable Cox Regression

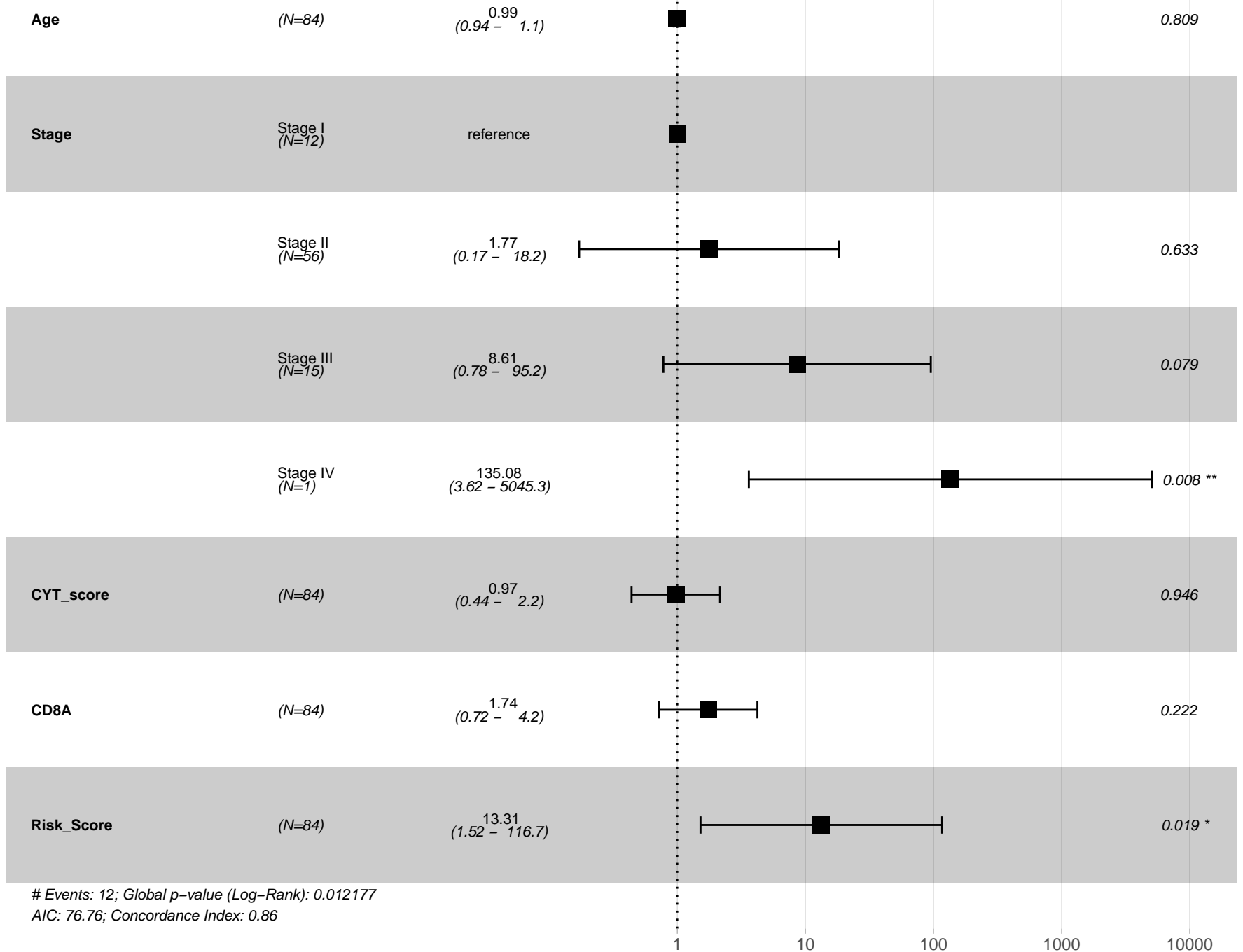

Supplement: Supplementary file 1 [file DataSheet1.zip › Supplement/Supplementary Figure S8.pdf]

Distribution of signature activity scores

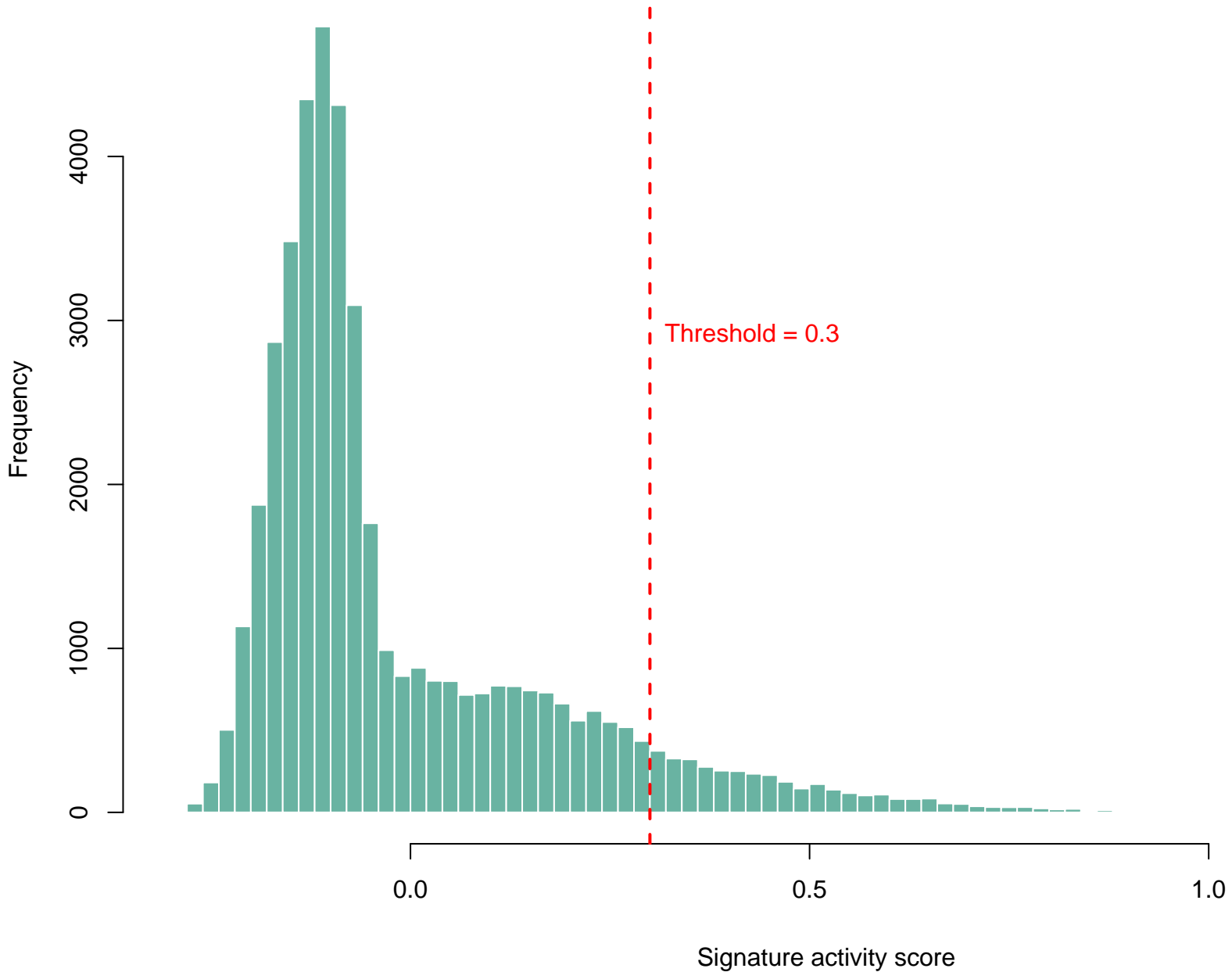

Supplement: Supplementary file 1 [file DataSheet1.zip › Supplement/Supplementary Figure S9.pdf]
